# Digital AVATAR therapy for distressing voices in psychosis: the phase 2/3 AVATAR2 trial

**DOI:** 10.1038/s41591-024-03252-8

**Published:** 2024-10-28

**Authors:** Philippa A. Garety, Clementine J. Edwards, Hassan Jafari, Richard Emsley, Mark Huckvale, Mar Rus-Calafell, Miriam Fornells-Ambrojo, Andrew Gumley, Gillian Haddock, Sandra Bucci, Hamish J. McLeod, Jeffrey McDonnell, Moya Clancy, Michael Fitzsimmons, Hannah Ball, Alice Montague, Nikos Xanidis, Amy Hardy, Thomas K. J. Craig, Thomas Ward

**Affiliations:** 1 Department of Psychology, Institute of Psychiatry, Psychology & Neuroscience, King’s College London, London, UK; 2 South London & Maudsley NHS Foundation Trust, London, UK; 3 Department of Biostatistics and Health Informatics, Institute of Psychiatry, Psychology and Neuroscience, King’s College London, London, UK; 4 University College London, London, UK; 5 Mental Health Research and Treatment Center, Faculty of Psychology, Ruhr-Universität Bochum, Bochum, Germany; 6 Research Department of Clinical, Educational and Health Psychology, University College London, London, UK; 7 North East London NHS Foundation Trust, London, UK; 8 School of Health and Wellbeing, University of Glasgow, Glasgow, UK; 9 NHS Greater Glasgow & Clyde, Glasgow, UK; 10 Division of Psychology and Mental Health, School of Health Sciences, University of Manchester and the Manchester Academic Health Sciences Centre, Manchester, UK; 11 Greater Manchester Mental Health NHS Foundation Trust and the Manchester Academic Health Sciences Centre, Manchester, UK; 12 Department of Health Service and Population Research, Institute of Psychiatry, Psychology & Neuroscience, King’s College London, London, UK

**Keywords:** Outcomes research, Randomized controlled trials

## Abstract

Distressing voices are a core symptom of psychosis, for which existing treatments are currently suboptimal; as such, new effective treatments for distressing voices are needed. AVATAR therapy involves voice-hearers engaging in a series of facilitated dialogues with a digital embodiment of the distressing voice. This randomized phase 2/3 trial assesses the efficacy of two forms of AVATAR therapy, AVATAR-Brief (AV-BRF) and AVATAR-Extended (AV-EXT), both combined with treatment as usual (TAU) compared to TAU alone, and conducted an intention-to-treat analysis. We recruited 345 participants with psychosis; data were available for 300 participants (86.9%) at 16 weeks and 298 (86.4%) at 28 weeks. The primary outcome was voice-related distress at both time points, while voice severity and voice frequency were key secondary outcomes. Voice-related distress improved, compared with TAU, in both forms at 16 weeks but not at 28 weeks. Distress at 16 weeks was as follows: AV-BRF, effect −1.05 points, 96.5% confidence interval (CI) = −2.110 to 0, *P* = 0.035, Cohen’s *d* = 0.38 (CI = 0 to 0.767); AV-EXT −1.60 points, 96.5% CI = −3.133 to −0.058, *P* = 0.029, Cohen’s *d* = 0.58 (CI = 0.021 to 1.139). Distress at 28 weeks was: AV-BRF, −0.62 points, 96.5% CI = −1.912 to 0.679, *P* = 0.316, Cohen’s *d* = 0.22 (CI = −0.247 to 0.695); AV-EXT −1.06 points, 96.5% CI = −2.700 to 0.586, *P* = 0.175, Cohen’s *d* = 0.38 (CI = −0.213 to 0.981). Voice severity improved in both forms, compared with TAU, at 16 weeks but not at 28 weeks whereas frequency was reduced in AV-EXT but not in AV-BRF at both time points. There were no related serious adverse events. These findings provide partial support for our primary hypotheses. AV-EXT met our threshold for a clinically significant change, suggesting that future work should be primarily guided by this protocol. ISRCTN registration: ISRCTN55682735.

## Abstract (web summary)

A randomized controlled trial involving people with psychosis who hear distressing voices reported that two different forms of AVATAR therapy, an intervention where voice-hearers engage with a digital avatar representing this distressing voice, reported further improvements in voice-related distress at 16 weeks but not at 28 weeks on top of standard of care.

## Main

Digital innovation carries the promise of transforming mental health treatment, addressing long-standing issues in access, engagement and effectiveness^1,2^. Auditory verbal hallucinations (henceforth voices), commonly associated with a diagnosis of schizophrenia, are often distressing and impair quality of life. However, the response to pharmacological and psychological treatments is suboptimal^3,4^, highlighting the need for new interventions. AVATAR therapy is one such digital innovation, which targets voices^5^. It is part of a wave of relational approaches, informed by advances in theory, which position voice-hearing as an experience of social communication^6,7^. The defining aspect of AVATAR therapy is the digital embodiment of the distressing voice in the form of an avatar. Bespoke software enables the voice-hearer to customize how the avatar looks and sounds. Treatment is focused on a series of ‘face-to-face’ dialogues between the person and their avatar, supported by the therapist. The aim is to reduce voice-related distress and build empowerment in daily life.

A proof-of-concept study found that a six-session course of AVATAR therapy was safe, with positive effects on voice severity^5^. A previous fully powered single-site randomized controlled trial (AVATAR1) compared AVATAR therapy with supportive counseling^8^ and demonstrated a substantial reduction in the severity of voices in the AVATAR therapy group at 12 weeks. An independent pilot also reported feasibility and efficacy findings^9^. Examination of AVATAR1 therapy content identified a wide range of potential treatment targets, including developmental trauma^10^, suggesting that the intervention could be optimized through personalization to diverse voice-hearer experiences^11,12^. Early evidence for AVATAR therapy is based on delivery by a small and experienced cohort of therapists within research settings. There is consequently a need to test effectiveness when treatment is delivered by a wider workforce, across geographically and demographically diverse locations, including frontline mental health services.

The main objective of this late phase 2/3 multisite AVATAR2 trial is to test, compared with treatment as usual (TAU) alone, the efficacy of two forms of AVATAR therapy, that is, AVATAR-Brief (AV-BRF), with a standardized focus on exposure, assertiveness and self-esteem, and AVATAR-Extended (AV-EXT), with a phase 1 mirroring AV-BRF, augmented by a more personalized, developmentally focused phase 2 based on the voice-hearer’s life history. We hypothesized that both AV-BRF and AV-EXT, when added to TAU, would be superior to TAU alone at 16 and 28 weeks in reducing voice-related distress (primary outcome), frequency and severity (key secondary outcomes).

## Results

### Patient disposition

**Fig. 1:**
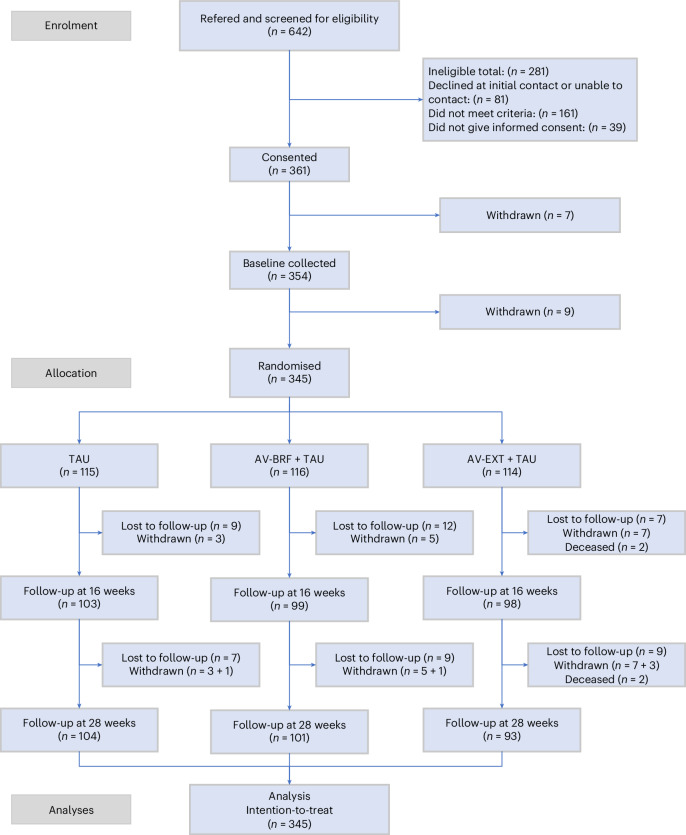
CONSORT diagram of all participants who were assessed for eligibility for the trial, randomized to AV-EXT + TAU, AV-BRF + TAU or TAU alone, and followed up to 28 weeks. Follow-up at 16 weeks: 16 weeks after baseline (post-treatment follow-up). Follow-up at 28 weeks: 28 weeks after the baseline.

Between 1 January 2021 and 30 November 2022, we assessed 642 people for eligibility, recruiting 345 participants and randomly allocating them to AV-BRF (*n* = 116), AV-EXT (*n* = 114) and TAU control (*n* = 115). Data were available for 300 participants at the 16-week follow-up (86.9%) and 298 (86.4%) at 28 weeks; at the 16-week follow-up, 12 participants were lost in TAU, 17 in AV-BRF and 16 in AV-EXT; the numbers lost were 11 (TAU), 15 (AV-BRF) and 21 (AV-EXT) at 28 weeks (see Fig. 1 for the participant Consolidated Standards of Reporting Trials (CONSORT) diagram).

The participants’ baseline demographic and clinical characteristics showed no differences between trial arms at baseline (Table 1). As is typical in a sample of people with psychosis, overall there was a greater proportion who were male (61.4%); most were single, unemployed and the most common diagnosis was schizophrenia (43.8%). Participants had been in contact with mental health services for an average of approximately 13 years (mean = 13.33, s.d. = 11.15), and approximately 40% belonged to a minoritized ethnic group. Their voices were assessed at baseline on the Psychotic Symptoms Rating Scale-Auditory Hallucinations (PSYRATS-AH) scale as similar in severity to those in the AVATAR1 trial^8^, with high mean scores for voice severity^13^. On average, 61.2% reported highly characterized voices.

**Table 1:** Demographic and clinical characteristics of the ITT population across trial arms at baseline

	Trial arm			
	TAU	AV-BRF	AV-EXT	Total
*n*	115 (33.3%)	116 (33.6%)	114 (33.0%)	345 (100.0%)
Age (years)	38.69 (±12.78)	39.35 (±13.31)	40.81 (±13.69)	39.61 (±13.26)
Age when they first started to hear voices (years)	24.39 (±11.99)	23.70 (±10.88)	25.93 (±12.10)	24.67 (±11.67)
Duration of contact with mental health services (years)	12.75 (±10.15)	13.24 (±11.61)	14.03 (±11.69)	13.33 (±11.15)
Gender				
Male	69 (60.0%)	72 (62.1%)	71 (62.3%)	212 (61.4%)
Female	44 (38.3%)	43 (37.1%)	42 (36.8%)	129 (37.4%)
Other	2 (1.7%)	1 (0.9%)	1 (0.9%)	4 (1.2%)
Ethnicity				
White	69 (60.0%)	63 (54.3%)	71 (62.3%)	203 (58.8%)
Black or mixed Black	19 (16.5%)	19 (16.4%)	19 (16.7%)	57 (16.5%)
South Asian or mixed South Asian	12 (10.4%)	9 (7.8%)	6 (5.3%)	27 (7.8%)
Other	15 (13.0%)	25 (21.6%)	18 (15.8%)	58 (16.8%)
Marital status				
Single	86 (74.8%)	87 (75.0%)	86 (75.4%)	259 (75.1%)
In a relationship	4 (3.5%)	11 (9.5%)	7 (6.1%)	22 (6.4%)
Cohabiting	10 (8.7%)	1 (0.9%)	3 (2.6%)	14 (4.1%)
Married or civil partnership	6 (5.2%)	8 (6.9%)	6 (5.3%)	20 (5.8%)
Divorced	9 (7.8%)	7 (6.0%)	12 (10.5%)	28 (8.1%)
Widowed	0 (0%)	2 (1.7%)	0 (0%)	2 (0.6%)
Living status				
Living alone (± children)	54 (47.0%)	54 (46.6%)	51 (44.7%)	159 (46.1%)
Living with husband/wife (± children)	5 (4.3%)	7 (6.0%)	5 (4.4%)	17 (4.9%)
Living together as a couple (± children)	10 (8.7%)	5 (4.3%)	6 (5.3%)	21 (6.1%)
Living with parents	31 (27.0%)	33 (28.4%)	31 (27.2%)	95 (27.5%)
Living with other relatives	4 (3.5%)	3 (2.6%)	6 (5.3%)	13 (3.8%)
Living with others	10 (8.7%)	12 (10.3%)	15 (13.2%)	37 (10.7%)
Not available or not applicable	1 (0.9%)	1 (0.9%)	0 (0%)	2 (0.6%)
Unknown	0 (0%)	1 (0.9%)	0 (0%)	1 (0.3%)
Highest level of schooling				
Primary school	1 (0.9%)	2 (1.7%)	2 (1.8%)	5 (1.4%)
Secondary, no exams qualifications	10 (8.7%)	9 (7.8%)	6 (5.3%)	25 (7.2%)
Secondary, O level or CSE equivalent	24 (20.9%)	20 (17.2%)	32 (28.1%)	76 (22.0%)
Secondary, A level equivalent	20 (17.4%)	14 (12.1%)	16 (14.0%)	50 (14.5%)
Vocational education or college	21 (18.3%)	35 (30.2%)	23 (20.2%)	79 (22.9%)
University degree or professional qualification	39 (33.9%)	34 (29.3%)	34 (29.8%)	107 (31.0%)
Not available or not applicable	0 (0%)	2 (1.7%)	1 (0.9%)	3 (0.9%)
Employment status				
Unemployed	85 (73.9%)	86 (74.1%)	86 (75.4%)	257 (74.5%)
Employed full-time	8 (7.0%)	12 (10.3%)	10 (8.8%)	30 (8.7%)
Employed part-time	8 (7.0%)	3 (2.6%)	5 (4.4%)	16 (4.6%)
Self-employed	0 (0%)	0 (0%)	2 (1.8%)	2 (0.6%)
Retired	1 (0.9%)	4 (3.4%)	2 (1.8%)	7 (2.0%)
Student	11 (9.6%)	9 (7.8%)	7 (6.1%)	27 (7.8%)
Housewife or husband	2 (1.7%)	2 (1.7%)	1 (0.9%)	5 (1.4%)
Not available or not applicable	0 (0%)	0 (0%)	1 (0.9%)	1 (0.3%)
Diagnoses (according to the ICD-10 codes)				
F20—Schizophrenia	54 (47.0%)	52 (44.8%)	45 (39.5%)	151 (43.8%)
F22—Persistent delusional disorders	2 (1.7%)	1 (0.9%)	0 (0%)	3 (0.9%)
F23—Acute and transient psychotic disorders	2 (1.7%)	0 (0%)	2 (1.8%)	4 (1.2%)
F24—Induced delusional disorder	0 (0%)	1 (0.9%)	0 (0%)	1 (0.3%)
F25—Schizoaffective disorders	6 (5.2%)	9 (7.8%)	12 (10.5%)	27 (7.8%)
F28—Other nonorganic psychotic disorders	4 (3.5%)	3 (2.6%)	1 (0.9%)	8 (2.3%)
F29—Unspecified nonorganic psychosis	31 (27.0%)	35 (30.2%)	41 (36.0%)	107 (31.0%)
F31—Bipolar affective disorder	3 (2.6%)	1 (0.9%)	4 (3.5%)	8 (2.3%)
F32.3—Severe depressive episode with psychotic symptoms	11 (9.6%)	14 (12.1%)	8 (7.0%)	33 (9.6%)
Not available or not applicable	2 (1.7%)	0 (0%)	1 (0.9%)	3 (0.9%)
Deprivation Index				
Most deprived	41 (35.7%)	54 (46.6%)	45 (39.5%)	140 (40.6%)
Second quintile	38 (33.0%)	33 (28.4%)	37 (32.5%)	108 (31.3%)
Third quintile	21 (18.3%)	13 (11.2%)	20 (17.5%)	54 (15.7%)
Fourth quintile	4 (3.5%)	6 (5.2%)	5 (4.4%)	15 (4.3%)
Least deprived	7 (6.1%)	8 (6.9%)	4 (3.5%)	19 (5.5%)
Unknown	4 (3.5%)	2 (1.7%)	3 (2.6%)	9 (2.6%)
PSYRATS-AH-Distress	15.70 (±2.78)	15.72 (±2.72)	15.89 (±2.77)	15.77 (±2.75)
PSYRATS-AH-Frequency	7.87 (±1.95)	7.39 (±2.11)	7.06 (±2.02)	7.44 (±2.05)
PSYRATS-AH-Total	30.64 (±4.42)	30.09 (±4.66)	30.11 (±4.42)	30.28 (±4.50)
Voice characterization				
More highly characterized (higher)	69 (60.0%)	71 (61.2%)	71 (62.3%)	211 (61.2%)
Less highly characterized (lower)	46 (40.0%)	45 (38.8%)	43 (37.7%)	134 (38.8%)

Values are presented as *n* (*n*%), indicating the frequency and its corresponding percentage of the total, while *n* (±*n*) indicates the mean value and s.d. ICD-10, International Statistical Classification of Diseases and Related Health Problems, 10th Revision.

### Treatment completion

A total of 95 of 116 (81.90%) participants assigned to the AV-BRF and 66 of 114 (57.89%) assigned to the AV-EXT completed treatment (against prespecified criteria, that is, four of six active sessions for AV-BRF and ten of 12 sessions for AV-EXT). Four (3.45%) participants allocated to AV-BRF and 37 (32.46%) to AV-EXT had partial treatment but did not reach the completion criterion. Seventeen people (14.66%) allocated to AV-BRF and 11 to AV-EXT (9.65%) attended no treatment sessions. For AV-BRF, the overall mean number of sessions attended was 5.11 (s.d. = 2.42; range = 0–8). For those who completed treatment, the mean was 6.16 sessions (s.d. = 0.94; range = 4–8). The mean active treatment session duration was 65.65 min (s.d. = 13.97, minimum = 30; maximum = 148), including a mean avatar dialogue duration of 9.51 min (s.d. = 3.79, minimum = 4, maximum = 28). For AV-EXT, the overall mean number of sessions was 8.18 (s.d. = 4.43; minimum = 0; maximum = 13). For those who completed treatment, the mean was 11.53 sessions (s.d. = 0.92; range = 10–13). The active treatment session time was 65.93 min (s.d. = 13.34; minimum = 20; maximum = 122), including a mean active dialogue duration of 10.47 min (s.d. = 3.95; minimum = 1; maximum = 27) (see Supplementary Materials 1–4 for additional data on treatment completion and mode of delivery).

### Primary outcomes

**Fig. 2:**
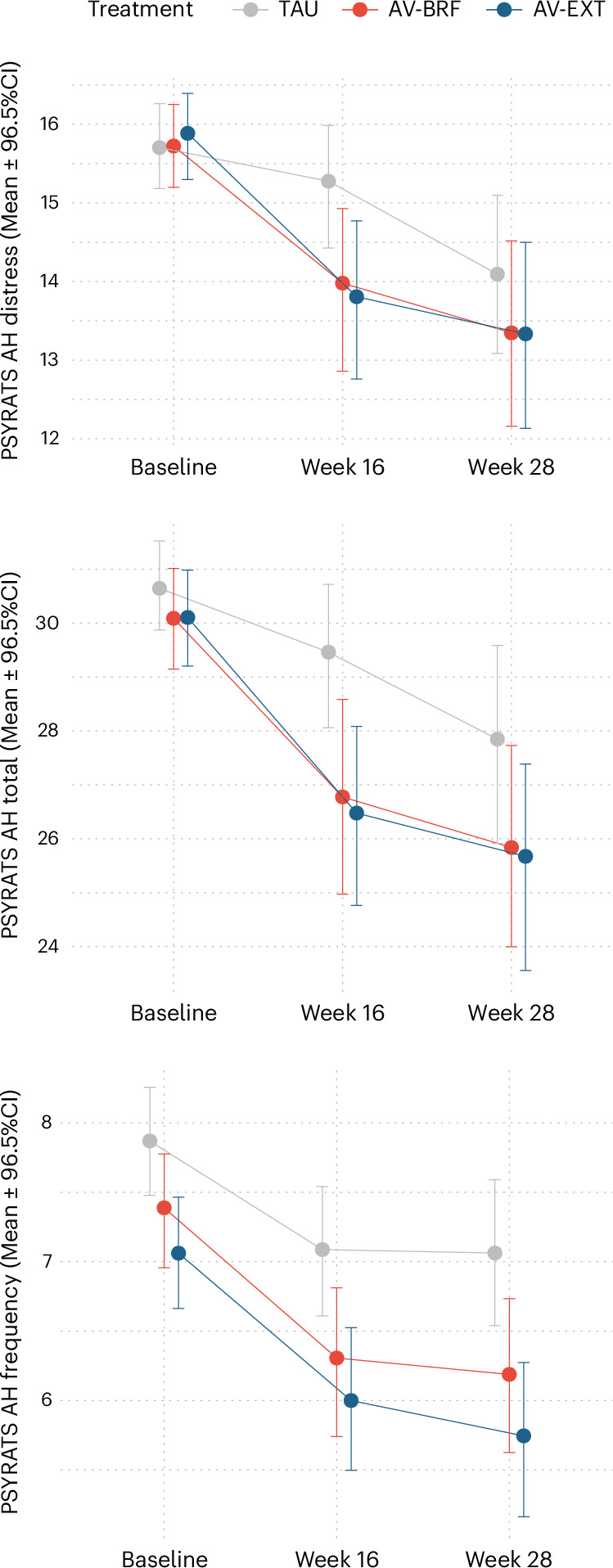
PSYRATS-AH-Distress, Total Severity and Frequency observed mean scores with 96.5% CIs. Week 16: 16 weeks after baseline (post-treatment follow-up). Week 28: 28-week follow-up after baseline. The center points represent the mean values with the 96.5% CIs. The sample size (*n*) for PSYRATS-AH-Distress, Total Severity and Frequency was 345 at baseline, 299 at week 16 and 298 at week 28.

As shown in Table 2 and Fig. 2, there was an improvement in voice-related distress on the distress subscale of the PSYRATS-AH-Distress (range = 0–20) in both forms at 16 weeks but not at 28 weeks (distress at 16 weeks: AV-BRF, effect −1.05 points, 96.5% confidence interval (CI) = −2.110 to 0, *P* = 0.035, Cohen’s *d* = 0.38 (CI = 0 to 0.767); AV-EXT, −1.60 points, 96.5% CI = −3.133 to −0.058, *P* = 0.029, Cohen’s *d* = 0.58 (CI = 0.021 to 1.139)). Distress at 28 weeks was as follows: AV-BRF, −0.62 points, 96.5% CI = −1.912 to 0.679, *P* = 0.316, Cohen’s *d* = 0.22 (CI = −0.247 to 0.695); AV-EXT −1.06 points, 96.5% CI = −2.700 to 0.586, *P* = 0.175, Cohen’s *d* = 0.38 (CI = −0.213 to 0.981). In the mixed-effects analysis of the primary outcome, the intraclass correlation coefficient (ICC) for the therapist clustering effect in both AV-EXT and AV-BRF was 0.054, indicating that approximately 5.4% of the residual variance in PSYRATS-AH-Distress was at the therapist level.

**Table 2:** Treatment effect estimates on primary and secondary outcomes

Outcome	*n*	Time	Comparison	Effect	s.e.	*P*	96.5% CI		Effect size
Primary outcome									
Voice-related distress									
PSYRATS-AH-Distress	314	16	TAU versus AV-BRF	−1.05	0.500	**0.035**	−2.110	0	0.38
			TAU versus AV-EXT	−1.60	0.729	**0.029**	−3.133	−0.058	0.58
		28	TAU versus AV-BRF	−0.62	0.615	0.316	−1.912	0.679	0.22
			TAU versus AV-EXT	−1.06	0.779	0.175	−2.700	0.586	0.38
Key secondary outcomes									
Voice frequency									
PSYRATS-AH-Frequency	314	16	TAU versus AV-BRF	−0.50	0.244	0.042	−1.012	0.018	0.24
			TAU versus AV-EXT	−0.62	0.246	**0.011**	−1.140	−0.104	0.30
		28	TAU versus AV-BRF	−0.65	0.323	0.044	−1.331	0.030	0.32
			TAU versus AV-EXT	−0.89	0.300	**0.003**	−1.525	−0.258	0.43
Voice severity									
PSYRATS-AH Total	314	16	TAU versus AV-BRF	−2.04	0.853	**0.017**	−3.836	−0.239	0.45
			TAU versus AV-EXT	−2.32	0.894	**0.009**	−4.208	−0.438	0.52
		28	TAU versus AV-BRF	−1.61	1.256	0.199	−4.260	1.036	0.36
			TAU versus AV-EXT	−1.87	1.138	0.100	−4.274	0.526	0.42
Other secondary outcomes									
Other voice-specific measures									
BAVQ									
Omnipotence	298	16	TAU versus AV-BRF	−0.63	0.456	0.167	−1.592	0.332	−0.18
			TAU versus AV-EXT	−0.72	0.652	0.270	−2.095	0.655	−0.20
		28	TAU versus AV-BRF	−0.82	0.402	0.042	−1.662	0.031	−0.23
			TAU versus AV-EXT	−1.28	0.589	**0.030**	−2.525	−0.040	−0.36
Malevolence	298	16	TAU versus AV-BRF	−0.44	0.469	0.352	−1.426	0.552	−0.10
			TAU versus AV-EXT	−0.38	0.571	0.507	−1.584	0.826	−0.09
		28	TAU versus AV-BRF	0.14	0.480	0.772	−0.873	1.152	0.03
			TAU versus AV-EXT	−0.26	0.711	0.712	−1.760	1.236	−0.06
Benevolence	298	16	TAU versus AV-BRF	0.11	0.367	0.767	−0.665	0.882	0.03
			TAU versus AV-EXT	0.52	0.417	0.212	−0.359	1.402	0.13
		28	TAU versus AV-BRF	0	0.435	0.993	−0.921	0.913	0
			TAU versus AV-EXT	0.67	0.505	0.186	−0.397	1.731	0.17
Total	298	16	TAU versus AV-BRF	3.05	1.548	0.049	−0.214	6.313	0.26
			TAU versus AV-EXT	3.00	1.248	**0.016**	0.372	5.634	0.26
		28	TAU versus AV-BRF	1.71	1.641	0.296	−1.747	5.175	0.15
			TAU versus AV-EXT	3.01	1.819	0.098	−0.824	6.846	0.26
VAAS									
Acceptance	297	16	TAU versus AV-BRF	3.41	0.815	**<0.001**	1.688	5.125	0.49
			TAU versus AV-EXT	3.88	1.048	**<0.001**	1.672	6.089	0.56
		28	TAU versus AV-BRF	2.84	0.813	**<0.001**	1.126	4.555	0.41
			TAU versus AV-EXT	3.98	1.196	**0.001**	1.458	6.500	0.58
Action	297	16	TAU versus AV-BRF	1.98	0.813	**0.015**	0.262	3.689	0.22
			TAU versus AV-EXT	3.26	0.974	**0.001**	1.205	5.313	0.36
		28	TAU versus AV-BRF	2.25	0.924	**0.015**	0.297	4.194	0.25
			TAU versus AV-EXT	2.83	1.042	**0.007**	0.635	5.027	0.31
Full-scale	297	16	TAU versus AV-BRF	5.51	1.406	**<0.001**	2.550	8.477	0.39
			TAU versus AV-EXT	7.17	1.776	**<0.001**	3.420	10.910	0.51
		28	TAU versus AV-BRF	5.12	1.462	**<0.001**	2.039	8.204	0.36
			TAU versus AV-EXT	6.83	1.989	**0.001**	2.634	11.022	0.48
VPDS	298	16	TAU versus AV-BRF	−0.35	0.160	**0.030**	−0.686	−0.010	0.28
			TAU versus AV-EXT	−0.36	0.144	**0.013**	−0.659	−0.053	0.29
		28	TAU versus AV-BRF	−0.17	0.136	0.205	−0.460	0.115	0.14
			TAU versus AV-EXT	−0.31	0.137	**0.022**	−0.601	−0.026	0.25
Hallucinations Remission Score	314	16	TAU versus AV-BRF	−0.11	0.094	0.233	−0.311	0.086	0.22
			TAU versus AV-EXT	−0.21	0.108	0.050	−0.439	0.016	0.41
		28	TAU versus AV-BRF	−0.02	0.157	0.894	−0.351	0.310	0.04
			TAU versus AV-EXT	−0.06	0.127	0.654	−0.324	0.210	0.11
Distressing persecutory beliefs (delusions)									
PSYRATS-Delusions	279	16	TAU versus AV-BRF	−1.85	0.776	**0.017**	−3.490	−0.216	0.37
			TAU versus AV-EXT	−2.86	1.220	**0.019**	−5.433	−0.289	0.57
		28	TAU versus AV-BRF	−1.24	0.780	0.111	−2.890	0.401	0.25
			TAU versus AV-EXT	−3.41	1.448	**0.019**	−6.459	−0.355	0.68
Well-being and recovery									
WEMWBS	291	16	TAU versus AV-BRF	2.19	0.949	**0.021**	0.185	4.186	0.20
			TAU versus AV-EXT	6.83	2.028	**0.001**	2.555	11.107	0.63
		28	TAU versus AV-BRF	2.03	1.084	0.061	−0.251	4.321	0.19
			TAU versus AV-EXT	5.10	2.280	**0.025**	0.294	9.909	0.47
CHOICE	291	16	TAU versus AV-BRF	7.39	2.529	**0.003**	2.061	12.723	0.33
			TAU versus AV-EXT	9.44	2.260	**<0.001**	4.673	14.204	0.42
		28	TAU versus AV-BRF	5.67	2.404	**0.018**	0.598	10.737	0.25
			TAU versus AV-EXT	6.40	2.473	**0.010**	1.181	11.610	0.29
Mood, anxiety, trauma									
DASS									
Anxiety	293	16	TAU versus AV-BRF	−4.50	0.953	**<0.001**	−6.512	−2.495	0.44
			TAU versus AV-EXT	−4.07	0.856	**<0.001**	−5.879	−2.269	0.40
		28	TAU versus AV-BRF	−3.26	0.936	**<0.001**	−5.232	−1.287	0.32
			TAU versus AV-EXT	−2.07	1.268	0.103	−4.740	0.605	0.20
Depression	293	16	TAU versus AV-BRF	−3.76	1.111	**0.001**	−6.102	−1.416	0.33
			TAU versus AV-EXT	−4.19	1.252	**0.001**	−6.829	−1.548	0.37
		28	TAU versus AV-BRF	−2.66	1.168	**0.023**	−5.122	−0.196	0.24
			TAU versus AV-EXT	−1.90	1.505	0.207	−5.073	1.274	0.17
Stress	293	16	TAU versus AV-BRF	−3.97	1.004	**<0.001**	−6.083	−1.848	0.39
			TAU versus AV-EXT	−3.57	1.239	**0.004**	−6.187	−0.962	0.35
		28	TAU versus AV-BRF	−3.57	0.965	**<0.001**	−5.606	−1.536	0.35
			TAU versus AV-EXT	−1.75	1.360	0.198	−4.616	1.118	0.17
BDI	291	16	TAU versus AV-BRF	−2.06	1.517	0.174	−5.261	1.137	0.14
			TAU versus AV-EXT	−3.52	1.665	**0.035**	−7.026	−0.005	0.24
		28	TAU versus AV-BRF	−1.73	1.547	0.264	−4.991	1.534	0.12
			TAU versus AV-EXT	−1.72	2.160	0.425	−6.279	2.831	0.12
ITQ									
DSO	257	16	TAU versus AV-BRF	−1.58	0.770	0.041	−3.199	0.046	−0.25
			TAU versus AV-EXT	−1.39	0.947	0.142	−3.387	0.606	−0.22
PTSD	259	16	TAU versus AV-BRF	−0.82	1.050	0.433	−3.038	1.391	−0.12
			TAU versus AV-EXT	−0.73	0.844	0.387	−2.512	1.049	−0.10
Anxiety (ESM)	139	16	TAU versus AV-BRF	−1.07	0.689	0.122	−2.520	0.387	0.25
			TAU versus AV-EXT	−2.10	0.731	**0.004**	−3.645	−0.563	0.50
		28	TAU versus AV-BRF	0.09	0.836	0.913	−1.671	1.855	0.02
			TAU versus AV-EXT	−0.29	0.861	0.734	−2.109	1.523	0.07

Effects are estimates of between-group mean difference after adjusting for site, voice characterization and baseline measurement of each outcome. DSO, disturbances in self-organization; PTSD, posttraumatic stress disorder; VAAS, Voices Acceptance and Action Scale. Time points: 16, 16 weeks after baseline (post-treatment follow-up); 28, 28 week follow-up after baseline. *n* represents the sample size for the longitudinal mixed model for each measure in the analysis. Bold denotes *P* ≤ 0.035.

### Key secondary outcomes

There was an improvement in PSYRATS-AH-Total voice severity (range = 0–44) in both forms at 16 weeks (AV-BRF, −2.04 points, 96.5% CI = −3.836 to −0.239, *P* = 0.017, Cohen’s *d* = 0.45 (CI = 0.053 to 0.853); AV-EXT −2.32 points, 96.5% CI = −4.208 to −0.438, *P* = 0.009, Cohen’s *d* = 0.52 (CI = 0.097 to 0.936)) but not at 28 weeks (AV-BRF, −1.61 points, 96.5% CI = −4.260 to 1.036, *P* = 0.199, Cohen’s *d* = 0.36 (CI = −0.230 to 0.947); AV-EXT −1.87 points, 96.5% CI = −4.274 to 0.526, *P* = 0.100, Cohen’s *d* = 0.42 (CI = −0.117 to 0.950)).

Voice frequency as measured by the PSYRATS-AH-Frequency subscale (range = 0–12) was significantly reduced in AV-EXT at both time points (16 weeks: −0.62 points, 96.5% CI = −1.140 to −0.104, *P* = 0.011, Cohen’s *d* = 0.30 (CI = 0.051 to 0.556); 28 weeks: −0.89 points, 96.5% CI = −1.525 to −0.258, *P* = 0.003, Cohen’s *d* = 0.43 (CI = 0.126 to 0.744)). Frequency was not reduced by AV-BRF at either time point (16 weeks: −0.50 points, 96.5% CI =−1.012 to 0.018, *P* = 0.042, Cohen’s *d* = 0.24 (CI = −0.009 to 0.494); 28 weeks: −0.65 points, 96.5% CI = −1.331 to 0.030, *P* = 0.044, Cohen’s *d* = 0.32 (CI = −0.015 to 0.649)).

### Other secondary outcomes

**Fig. 3:**
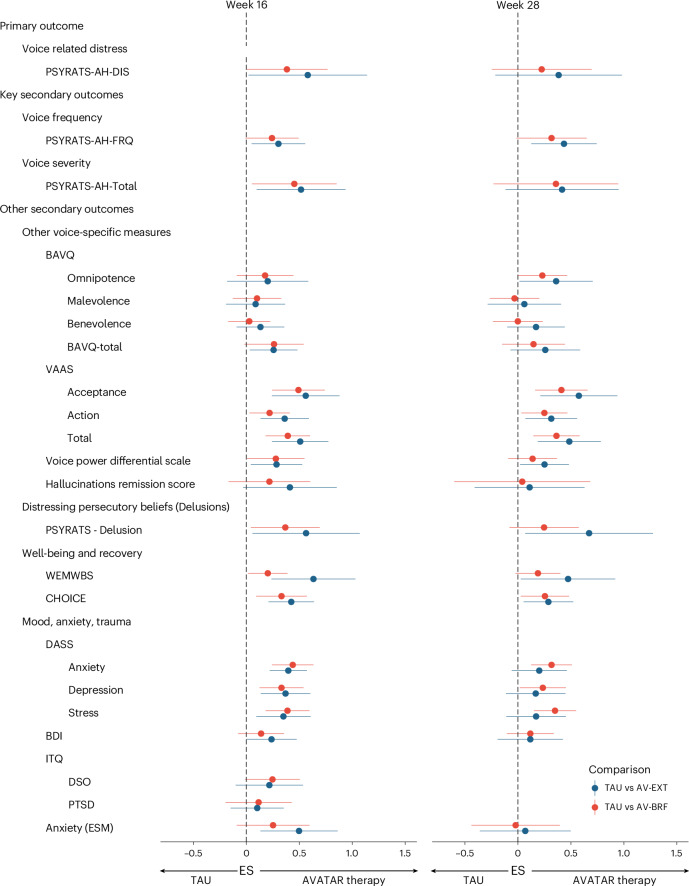
Effect size estimates with 96.5% CIs for primary and secondary outcomes at 16 and 28 weeks. Week 16: 16 weeks after baseline (post-treatment follow-up). Week 28: follow-up 28 weeks after baseline. The effect sizes (center points) with 96.5% CIs (error bars) for each outcome are shown. Effect sizes were calculated by dividing the estimated treatment effects from the mixed model and its 96.5% CI by the baseline s.d. of that outcome. The sample size (*n*) for each mixed-model outcome is provided in Table 2.

Table 2 also shows the treatment effect estimates across all other secondary outcomes. For other voice-specific measures, there were improvements in voice acceptance and action for both AV-BRF and AV-EXT at both time points; for the Beliefs About Voices Questionnaire (BAVQ) malevolence or benevolence, there were no effects for AV-BRF or AV-EXT at either time point nor for omnipotence at 16 weeks, although there was an effect on omnipotence for AV-EXT only at 28 weeks; the Voice Power Differential Scale (VPDS) score improved in both arms at 16 weeks and in AV-EXT at 28 weeks; finally, there were no effects on the Hallucinations Remission Score in either arm at either time point. There were reductions in PSYRATS-Delusions and improvements in well-being (Warwick-Edinburgh Mental Well-being Scale (WEMWBS)) for AV-EXT at both time points and for AV-BRF at week 16. There were improvements in personal recovery (CHoice of Outcome In Cbt for PsychosEs (CHOICE)) for AV-EXT and AV-BRF at both time points, and reductions in anxiety, depression and stress (measured by the Depression, Anxiety and Stress Scale (DASS)) for AV-BRF at both time points but only at week 16 for AV-EXT. Depression measured by the Beck Depression Inventory (BDI) and anxiety in daily life measured with the experience sampling method (ESM) showed improvements at 16 but not 28 weeks in AV-EXT, but not AV-BRF. There were no effects on the International Trauma Questionnaire (ITQ) for either arm at either time point. Figure 3 summarizes the standardized mean differences on all outcomes at both time points (Extended Data Tables 1 and 2 give the descriptive statistics for all measures at each time point).

### Safety

Table 3 presents all serious adverse events (SAEs) according to arm and event type. There were 58 SAEs across 56 participants, with 51% of events occurring in the AV-EXT arm. Most events were admission to hospital for psychological health events and these occurred equally across arms. There were two deaths in the AV-EXT arm. One of the deaths was a suicide, which occurred in the context of a long-standing pattern of suicidality-related hospital admissions, with increased alcohol use identified as a key factor. The independent Data Monitoring and Ethic Committee (DMEC) deemed this to be unrelated to treatment. For the second death, it was not possible to establish a definite cause of death. However, a serious untoward incident review was conducted independently by the responsible NHS trust and concluded that there was no evidence of a relationship between the death and engagement with AVATAR therapy; therefore, it was determined to be unrelated to treatment or other trial procedures by the independent DMEC. No SAEs were related to trial procedures (treatment, device or assessment). Six events, involving five participants, were ‘possibly related’ to treatment. A ‘possibly related’ rating meant that the DMEC Chair did not determine that it was related but could not definitively rule out a relationship. The single ‘possibly related’ event for AV-BRF and four of the five for AV-EXT were hospital admissions (the other was a crisis team involvement). The main factor in the rating for each of these as ‘possibly related’ was the timing of the event being close to the AVATAR therapy course; however, in each case there were plausible unrelated contributory factors linked to admission identified by the clinical team and reviewed by the DMEC (for example, life stressors and substance use).

**Table 3:** Serious adverse events according to treatment type across the trial arms

	TAU P, E (%) 14,14 (24.1%)	AV-BRF P, E (%) 14, 14 (24.1%)	AV-EXT P, E (%) 28, 30 (51.7%)
Serious adverse events			
Distress associated with completion of assessment measures	–	–	–
Significant distress during AVATAR therapy	–	–	–
Admission to hospital for psychological health event	8, 8 (57.1%)	8, 8 (57.1%)	8, 9 (30.0%)
Admission to hospital for physical health event	1, 1 (7.1%)	2, 2 (14.3%)	5, 5 (16.7%)
Referral to crisis team	2, 2 (14.3%)	1, 1 (7.1%)	6, 6 (20.0%)
Violent incident necessitating police involvement (victim)	–	–	–
Violent incident necessitating police involvement (accused)	1, 1 (7.1%)	–	–
Deliberate self-harm	–	2, 2 (14.3%)	3, 4 (13.3%)
Other psychological health event	1, 1 (7.1%)	–	3, 3 (10.0%)
Other physical health event	1, 1 (7.1%)	1, 1 (7.1%)	1, 1 (3.3%)
Death	–	–	2, 2 (6.7%)

P, E (%) represents participant and events (% of events).

AV-EXT showed a higher number of AEs compared to the two other arms. The category ‘Other physical health event’ contributed to this elevated number; it is unlikely to be treatment-specific in cause. Additional data tables are given in Supplementary Tables 5–8.

### Moderation and compliance-adjusted analysis

We tested for moderation of treatment effects in a prespecified set of putative baseline moderators. There was no moderation according to low or high voice characterization for either comparison at either time point. The only moderation effects to meet the significance threshold related to comparisons for AV-EXT between Index of Multiple Deprivation quintiles (Q2 versus Q1 at 16 and 28 weeks; Q4 versus Q1 at 16 weeks) and an interaction for age at first hearing voices for AV-BRF, where an earlier onset of AHs may have been associated with larger treatment effects (Extended Data Tables 3 and 4). However, given the number of statistical tests, these findings may have occurred by chance. We estimated complier average causal effects (CACEs) using two definitions of treatment compliance and estimated larger CACE than intention-to-treat (ITT) effects for most comparisons. The overall pattern of findings is similar to that of the primary analysis, with larger between-group effects at 16 weeks and no between-group differences at 28 weeks (Extended Data Table 5).

## Discussion

This multisite randomized trial of AVATAR therapy, investigated brief and extended forms, and tested delivery by a large cohort of therapists across geographically diverse sites. Voice distress mean scores and overall mean voice severity significantly improved in both AV-BRF and AV-EXT at end of treatment (16 weeks), compared to TAU alone. These improvements were maintained at 28 weeks, although they were no longer statistically significant. Voice frequency was reduced by AV-EXT (but not by AV-BRF) compared to TAU at the end of treatment (16 weeks); this improvement was sustained at the follow-up (28 weeks). The finding that AV-EXT demonstrated sustained reduction in the frequency of the occurrence of voices is relevant to research that shows that voice reduction (or cessation) is a clear priority for service users^14^. In summary, these findings meet the criteria prespecified in our statistical analysis plan for partial support of our main hypotheses, which stated that both versions of AVATAR therapy would be superior to TAU alone, at post-treatment and at follow-up, in reducing voice-related distress, voice severity and voice frequency.

The between-group effect sizes (that is, each version of therapy versus TAU) on voice-related distress post-treatment had a Cohen’s *d* = 0.58 for AV-EXT and *d* = 0.38 for AV-BRF. These are greater than or equal to comparable post-treatment effect sizes of around 0.3–0.4 reported in recent meta-analyses for longer courses of cognitive behavioral therapy for psychosis (CBTp)^4,15^, the current psychological treatment recommended by the National Institute for Clinical and Health Excellence (NICE)^16^. While AV-EXT treatment exceeded the threshold we prespecified for a clinically significant post-treatment change (that is, an effect size of 0.5 standard deviation), AV-BRF was slightly below this level with an associated *P* value just at the prespecified threshold for statistical significance (*P* = 0.035), suggesting some caution in its interpretation.

Secondary outcomes included recognized priorities for voice-hearers^14^ (Supplementary Materials and Supplementary Table 9) and as such are worthy of some consideration. While the trial was not designed for a direct comparison between AV-BRF and AV-EXT, the different secondary outcome effects of each, compared to TAU alone, are also informative. AV-BRF delivered benefits at both time points (albeit with relatively small between-group effects) on important secondary outcomes, including personal recovery, voice acceptance and action and mood (anxiety, depression and stress), with the latter being the single domain where sustained effects were observed for AV-BRF but not AV-EXT. Engagement in AV-BRF was also strong, with treatment completion rates of over 80%. AV-EXT delivered benefits at both time points across well-being, personal recovery and empowerment, outcomes that were not demonstrated in earlier AVATAR studies^5,8^. There were also significant post-treatment between-group effects for AV-EXT on depression, stress and anxiety, with convergent evidence from ESM data for anxiety reduction in daily life. However, there were no significant differences for AV-EXT in mood outcomes at 28 weeks. With respect to the hypothesized effects on beliefs about voices, improvements were observed for AV-EXT in omnipotence but not malevolence. Finally, evidence of improvements in persecutory distressing beliefs linked to the voice showed a moderate-to-large effect size at 28 weeks (*d* = 0.68), which is approximately double the typical findings reported for delusions in meta-analyses of CBTp^4,15^. This evidence supports AV-EXT delivering important and sustained changes in the personal understanding of the voice, which were not observed in AV-BRF.

AVATAR therapy as delivered in this trial was safe. There were no SAEs rated by the independent DMEC as related to treatment or the medical device. There was a larger number of overall SAEs and adverse events (AEs) reported in AV-EXT, across a diverse range of categories; this is probably at least partially attributable to the opportunity for increased monitoring and reporting provided by therapists over more sessions in AV-EXT. In addition, the trauma focus within AV-EXT is a possible factor in the higher recording of affective changes (as AEs) that did not meet the threshold for SAEs (that is, transient increases in distress or voice-hearing that resolved over time). The findings from this multisite trial allied to those reported in the AVATAR1 trial provide evidence supporting the safety of AVATAR therapy.

Despite the range of significant sustained secondary outcomes, the lack of a significant effect on the primary outcome at 28 weeks in AV-EXT is to be acknowledged and considered. Treatment completion of just under 60% for AV-EXT (compared to just over 80% for AV-BRF) may be plausibly linked to the increased direct trauma-focused work within sessions and suggests the need for improved treatment engagement, adherence and, consequently, efficacy, based on learning from the current trial. Consistent with this, the compliance-adjusted analysis showed larger treatment effect estimates than the ITT findings in the subgroup of participants who complied with their allocation by fully completing treatment. Improved engagement may be delivered through use of a collaborative review around an optimal session number to ensure that the person retains a strong sense of control, particularly around trauma-focused work. Planned qualitative analysis, which will explore the experience of direct dialoguing with voice content and includes individuals who did not complete treatment, will inform this future optimization of treatment engagement. Another key challenge is how to optimize and sustain the real-world impact from the (often) powerful change in distress observed within dialogues. The current method (provision of complete dialogue recordings to listen to at home) was subject to variable engagement and could be enhanced. Work is soon to commence on innovation in artificial intelligence (AI)-powered virtual conversational agents capable of delivering avatar dialogues (Wellcome ref. no. 227721/Z/23/Z). In addition to boosting future scalability, AI integration with mobile technology would transform between-session practice, potentially boosting long-term efficacy. There is also interest in the use of immersive virtual reality to enhance AVATAR therapy delivery and effects. The evidence is currently limited, but the results of AVATAR VRSocial in Germany (ISRCTN35980117) and independent trials in Denmark and Canada will be informative^17,18^ Finally, we plan to examine hypotheses concerning the mechanisms of change in a future analysis, which will guide further refinement of the approach. Candidate mechanisms include reduced anxiety and sense of threat, and increased empowerment and voice acceptance.

Although previous work found that duration of AVATAR voice dialogues and everyday behavioral engagement with voices were related to more complex characterization^19^, the moderation analysis did not support our hypothesis that greater baseline complexity of voice characterization would moderate treatment effects. Furthermore, there were very few demographic variables that moderated treatment effects; given the number of tests, we caution that these findings may have occurred by chance. Overall, the results suggest no robust evidence of differential effectiveness for either AV-BRF or AV-EXT across clinical or demographic variables.

The design of this trial had some limitations. First, the use of a TAU control meant that we could not determine the benefits of AVATAR therapy compared to another psychological treatment. The current frontline psychological therapy (CBTp), recommended by NICE as a minimum of 16 sessions, is notably longer in duration than even AV-EXT. In our previous trial, we adapted a form of brief supportive counseling as a control of comparable duration, but this is not routinely available and was outperformed by AVATAR therapy; therefore, a TAU comparison was used for this larger, pragmatic, multisite study^20^. The ICC for the therapist clustering effect indicated that around 5% of the residual variance in primary outcome was at the therapist level. Based on our previous trial, which showed a smaller therapist ICC, we did not account for therapist clustering effects in the sample size calculation; however, this was considered within the analysis models. Finally, the trial was not fully powered for a comparison of AV-BRF with AV-EXT because the sample size required was impracticable. Health economic analysis, to be reported separately, will offer relevant information on the cost-effectiveness of both versions.

AVATAR therapy is one of several evidence-based digital health interventions emerging for psychosis and schizophrenia^21–24^. AVATAR therapy offers the experience of a powerful digital ‘sense of presence’ of a distressing voice, shared with the therapist and enabling rapid change and reduced frequency^12^. The delivery of the trial, across a geographically and demographically diverse sample and including therapists from a range of disciplines in routine clinical settings, strengthens the real-world relevance of these findings. In the context of adaptation to the coronavirus disease 2019 (COVID-19) pandemic, remote delivery of AVATAR therapy has been shown to be feasible and acceptable, which is promising for future scalability (see also the AMETHYST trial; ClinicalTrials.gov registration: NCT05982158). A recently published NICE-Early Value Assessment of digital therapies for psychosis recommended AVATAR therapy for NHS deployment while further real-world evidence is generated^25^. The data reported in this article on efficacy and safety provide evidence to inform this ongoing evaluation. Forthcoming trial outputs (to be reported separately) will provide cost-effectiveness analysis and include qualitative studies of diverse patient and clinician perspectives on AVATAR therapy. Building on the AVATAR2 data, real-world evidence of clinical and cost-effectiveness, safety and acceptability of AVATAR therapy when implemented in routine care is now required to support a full NICE submission and facilitate widespread NHS adoption.

In conclusion, this study has provided partial support for the primary hypothesis, in that there were superior effects on the primary outcome of voice-related distress of AVATAR therapy over TAU alone at 16 weeks, in both AV-BRF and AV-EXT versions, and that AV-EXT met our threshold for clinically significant change; however, the effects were no longer statistically significant at 28 weeks. In addition, we have provided indications of a wider range of sustained improvements in outcomes prioritized by voice-hearers, of the longer formulation-based AV-EXT version, which connects dialogues to the person’s life history^7^. Treatment completion of AV-BRF was high, while comparable rates for AV-EXT suggested the need for refinement to improve engagement. Based on these trial findings, we recommend that future development and provision of AVATAR therapy is primarily guided by the AV-EXT protocol.

## Methods

### Study design and oversight

This multisite, parallel-group, assessor-blinded, randomized controlled trial assessed the efficacy and safety of two forms of AVATAR therapy, AV-BRF (six sessions with a standardized focus on exposure, assertiveness and self-esteem) plus TAU or AV-EXT (12 sessions, with an initial phase mirroring AV-BRF followed by a personalized, developmentally focused second phase) plus TAU compared to TAU alone on reducing voice-related distress (primary outcome), voice-related frequency and severity (key secondary outcomes), and other mood, well-being and voice-related outcomes. The study received ethical approval (Camberwell St. Giles Research Ethics Committee: no. 20/LO/0657; Integrated Research Application System no. 277118) and was prospectively registered with the ISRCTN registry at which the published trial protocol^11^ and statistical analysis plan can also be accessed (ISRCTN55682735). The trial complied with the International Conference on Harmonization Good Clinical Practice guidelines and the 2013 Declaration of Helsinki. The study was overseen by an independent trial steering committee and a separate independent data monitoring and ethics committee. All participants provided written informed consent.

#### Trial protocol deviations

Before participant recruitment commenced, the COVID-19 pandemic began. Face-to-face contact was restricted intermittently. The study start was delayed for 3 months, as mandated nationally, and the protocol and procedures were adapted to allow for remote delivery of the trial. The final trial protocol (v.1.2) was approved before participant recruitment commenced and no changes were made to the protocol for the conduct of the trial subsequent to the start of the trial.

### Participants

Between 1 January 2021 and 30 November 2022, we assessed 642 people for eligibility, recruiting 345 participants. Participants were randomized at four study sites, each recruiting from two mental health service providers, in the United Kingdom (three in England: South London, North London, Manchester; one in Scotland: Glasgow) and were randomly allocated to three parallel arms: 116 to AV-BRF, 114 to AV-EXT and 115 to TAU control (Fig. 1).

Participants were referred by a clinician at the participating clinical sites. Other routes to participation included contact through institutional research registers or self-referral.

The inclusion criteria were as follows: (1) aged 18 years or over; (2) currently under the care of a specialist mental health team; (3) current frequent and distressing voices (as measured by a score of at least one on each of the intensity of distress and frequency items of the PSYRATS-AH (Voices) Scale^26^), persisting for at least 6 months and spoken in English; (4) speak and read English to a sufficient level to provide consent and complete the assessment procedures; (5) a clinical diagnosis of schizophrenia spectrum disorder (ICD-10 F20–29) or affective disorder with psychotic symptoms (ICD-10 F30–39, subcategories with psychotic symptoms) as determined through clinical records and additional consultation with the clinical team, if required. Criteria for exclusion included: (1) primary diagnosis of substance disorder, personality disorder or learning disability; (2) lacking capacity to consent; (3) profound visual or hearing impairment or insufficient comprehension of English to be able to engage in assessment or treatment; (4) currently undertaking individual psychological treatment for voices; (5) currently experiencing an acute mental health crisis.

### Randomization and masking

After baseline assessment, we randomly assigned (1:1:1) eligible participants via a secure independent web-based service hosted by the King’s Clinical Trials Unit, using randomly varying sized blocks (three and six), stratified according to site and baseline voice characterization (more or less) as defined by meeting the threshold for more highly characterized voices (score > 7) on the Voice Characterisation Checklist^27^.

Research assessors were masked to allocation and procedures were followed to maintain their masking (assessors did not have access to clinical records after the baseline (pre-randomization) assessment or access to the treatment database at any stage); all assessments were done at sites remote from the clinic and participants were reminded before each assessment not to disclose their allocation. It is not possible to mask psychological treatment participants or therapists to their allocation; site coordinators were unmasked and informed participants. Therapists were allocated at each site based on availability. Breaks in assessor masking were recorded; if unmasking occurred, reallocation to another rater occurred. All primary and key secondary outcomes (PSYRATS-AH Scale) were assessed by blinded assessors. Unmasking occurred in 29 assessments (8.4%) at 16 weeks and 15 assessments (4.3%) at 28 weeks. All assessments of these individuals were scored by blinded assessors after these instances of unmasking.

### Procedures

#### The intervention

AVATAR therapy is a digital treatment in which the person engages in face-to-face dialogues with a personalized digital embodiment of the voice (‘the avatar’). The avatar is presented to the person on a two-dimensional computer screen.

In the AVATAR2 trial, AVATAR therapy was delivered in two versions, according to the randomized condition, that is, AV-BRF and AV-EXT, according to a comprehensive clinical manual.

#### Therapy structure

Both versions commence with an initial clinical assessment session, which includes creation of the avatar. Approximately 20 min are dedicated to making the avatar of the person’s main distressing voice. This is to create a tangible representation of the voice, with a face, to whom the person can directly address their resistance. The aim is to create a voice and an image that is a ‘good enough’ representation of the voice for the person; the created avatar tends to achieve a surprisingly good match. However, in practice, there is a balance between creating a workable representation of the voice while ensuring that the person does not feel overburdened or pressured to achieve ‘a perfect match’—in this context, as a rule of thumb, 70% is considered a good match. Where possible the avatar should represent the dominant persecutory voice as identified by the person. While some may experience a rotating gallery of characters, the guiding principle is to create an avatar that best represents the group of voices and recommend that the person tries out what works with the avatar for other distressing voice(s) they experience.

**Extended Data Fig. 1:**
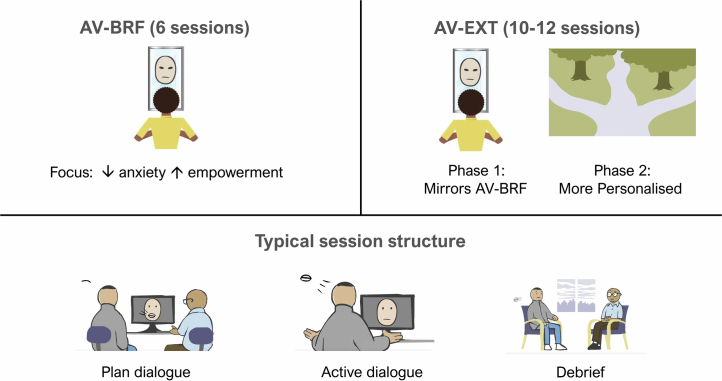
Illustration of AV-BRF and AV-EXT and typical structure for an active dialogue session. Treatment foci and session structure.

AV-BRF consists of six individual, face-to-face sessions delivered by trained therapists using the AVATAR therapy software. AV-BRF is designed to include the core aspects of AVATAR therapy, specifically the use of the avatar to deliver a realistic enactment of the voice (including exposure to verbatim voice content) and a treatment focus on increasing power and control and self-esteem. AV-EXT consists of 12 individual, face-to-face sessions and consists of two phases. The first phase mirrors AV-BRF. The aim of phase 2 is to develop an understanding of the voice(s) within the broader context of the person’s life and relationship history, informing a series of dialogues that flexibly target a wider range of treatment targets^10^ (Extended Data Fig. 1). For both versions, sessions could be increased or reduced by a maximum of two for treatment completion, guided by the clinical judgment of the therapist and in collaboration with the participant.

Each session (in both versions) consists of three parts: (1) pre-dialogue discussion; (2) active avatar dialogue; and (3) post-dialogue debrief. The whole takes 45–60 min. Pre-dialogues involve a review of the previous week (changes in the voice and other progress), identification of the main themes to be tackled in the forthcoming dialogue and, as necessary, practice role-play focused on the anticipated challenges within the dialogue. For AV-EXT, the pre-dialogue (particularly from the mid-treatment review onward) is also used to explore and formulate the possible contribution of previous traumatic experience to the voice-hearing experience, including instances of abuse, bullying, racial and sexual discrimination, or other forms of social exclusion and marginalization. During active dialogues, the therapist and the voice-hearer sit in separate rooms, communicating digitally, with the therapist remotely viewing the participant using a webcam. The post-dialogue session discusses the dialogue experience, commenting on the strengths shown by the person, discussing emerging content and finally giving a recording of the dialogue session and encouragement for the week ahead.

#### Therapy delivery

Therapy was intended to be delivered in person, at a participant’s local mental health clinic. However, because of the COVID-19 pandemic, the software was adapted to support remote treatment delivery using video conferencing software. This allowed participants to have treatment sessions from home, joining their therapist via remote web link. Eighty-seven percent of participants (*n* = 200) had treatment in person at the clinic (99 of 114 (87%) for AV-EXT and 101 of 116 (87%) for AV-BRF). Further data on face-to-face and remote delivery can be found in Supplementary Tables 3 and 4.

#### Therapist training

Of the 19 therapists who participated in the trial, 12 were qualified clinical or counseling psychologists, five were psychiatrists (three specialist trainee and two consultants) and two were nurse therapists; 11 were female and eight were male. The mean years of experience in delivering psychological treatment before commencing AVATAR therapy was 11.6 years (s.d. = 10.3, range = 1–40); 18 of 19 had more than 6 months’ experience of psychosis intervention at the start of their involvement in the trial. Two of the therapists were expert AVATAR therapists and trainers and delivered treatment in the previous AVATAR1 trial^8^. All other therapists were trained for the study. Training involved a combination of direct teaching and self-directed learning (including access to live treatment reference material), followed by closely supervised training cases. After the training period, the treatment supervision model included 1:1 (typically weekly) and group-based peer supervision (typically monthly). Sharing of live audio was a crucial aspect of supervision to inform discussions around the key treatment processes to be targeted (both in terms of enacting the avatar and suggested consolidation work before and after dialogue).

#### Adherence, fidelity and competence

Treatment adherence was assessed by the number of sessions attended. Fidelity to the clinical manual was assessed by the therapist completing a session-by-session checklist. An a priori checklist of therapist fidelity to protocolized components of treatment was developed based on earlier AVATAR clinical trials with specific additions for AV-EXT. Fidelity was predefined as completion of 80% of the specified components for each session. For both AV-BRF and AV-EXT, the mean self-reported fidelity for each session was more than 90%, with an overall mean rating (across all sessions) for AV-BRF of 92.46 (s.d. = 9.57; minimum = 19.64; maximum = 100) and for AV-EXT a mean of 93.38 (s.d. = 8.61; minimum = 17.31; maximum = 100) for AV-EXT.

Therapist competence was assessed by an expert in AVATAR therapy for both general and clinical and AVATAR-specific skills. Each newly trained trial therapist was rated for competence based on the review of early, mid and late session treatment delivery for at least one completed intervention. Ratings were conducted for two cases for therapists who delivered completed treatment with more than five participants. Cases were selected at random for each therapist, but excluding any cases where audio recordings were not available (for example, because of technical issues or the participant not consenting to a full recording). The rating tool was adapted from AVATAR1 to allow for different skill requirements for each level of treatment. For AVATAR-BRF, it included five items for session one and six (each) for mid and later sessions (17 items). The AVATAR-EXT rating tool mirrored this with the key difference being one additional item rated at the mid and last sessions to capture ‘promoting an understanding of voice within broader autobiographical and person-specific context’ (total items = 19). Each item was rated 1–5 with a total possible score across the three sessions of 85 for AVATAR-BRF (17 items) and 95 for AVATAR-EXT (19 items), with a benchmark of 3/5 per item for competent delivery (or 60% for the total score across all items). The mean competence rating for AV-BRF (*n* = 10 cases) was 79.8% (s.d. = 13.5); for AV-EXT (*n* = 13 cases), it was 76.8% (s.d. = 13.5).

#### AVATAR hardware and software

The Avatar Therapy System facilitates the delivery of AVATAR therapy for voice-hearing through a mix of commodity computer hardware and custom software. The software supports both enrollment of an avatar for the voice-hearer and real-time communication between the therapist and the voice-hearer using the avatar as a third party in a treatment session. The computing platform consists of two Windows laptops (or a laptop and desktop and a tablet) connected over a network. These can either be located within two rooms in the clinic (local delivery), or can be located at the therapist’s office and the client’s home (remote delivery). The key technical elements of the software include voice enrollment, face enrollment, real-time voice conversion, real-time lip synchronization and real-time animation.

**Extended Data Fig. 2:**
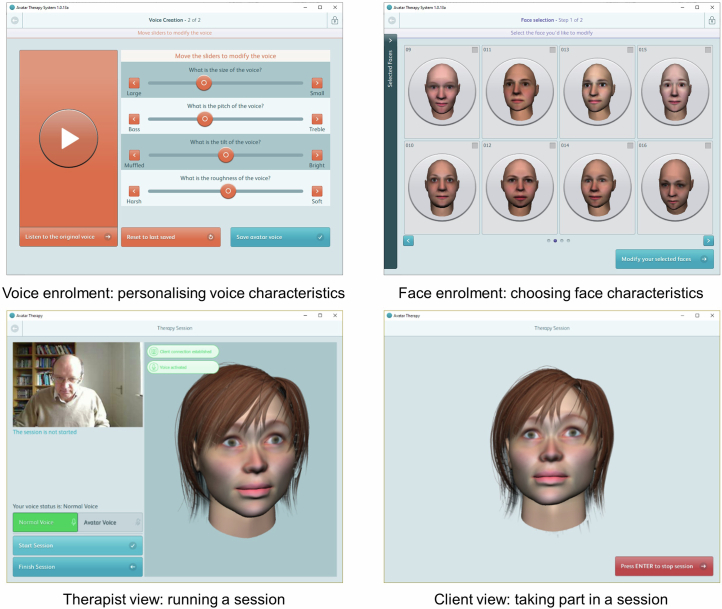
Illustration of AVATAR Therapy therapist and client interface. AVATAR therapy software interface.

Voice enrollment is the process by which the client chooses a voice for the avatar. The therapist makes a recording of the client’s normal voice and the software manipulates that voice along dimensions of pitch, vocal tract size, spectral tilt and temporal roughness. Slider controls on the interface allow the client to hear many different variations of the therapist’s voice until a good match to the ‘voice’ is found (Extended Data Fig. 2). These control settings are chosen and saved.

Face enrollment is the process by which the client chooses a face for the avatar. The underlying technology for creating and modifying faces is called FaceGen and is licensed from Singular Inversions. In face enrollment, a set of faces that match the basic attributes of the heard voice is generated and the closest one is chosen by the client. The software then allows the manipulation of facial shape, color and texture, as well as the addition of hair. The software supports different ethnicities and some nonhuman characters, such as a devil, witch and robot.

Real-time voice conversion is a technology for converting the therapist’s voice to the avatar’s voice within a live treatment session. The stored voice transformation settings chosen during voice enrollment are applied to the therapist’s voice recorded from a headset microphone; the converted voice is then communicated to the client’s computer over the network.

Real-time lip synchronization is the process by which representative mouth shapes and jaw positions of the avatar are chosen from an acoustic analysis of the speech signal being produced by the therapist when speaking as the avatar. This mapping between acoustic signal and visemes is performed by a neural network classifier.

Real-time animation is the process by which the three-dimensional model of the avatar is animated during the treatment session to make it look like the avatar is engaging in a dialogue. This is achieved by morphing the three-dimensional graphical model of the avatar according to the viseme output of the lip synchronization component while it is speaking. In addition, the avatar looks around and blinks occasionally so that it looks more alive.

The Avatar Therapy System also acts as a database of therapists, clients, avatars and sessions. It keeps recordings of treatment sessions, which can be shared with clients. Facilities also exist for backup and synchronization between a group of laptops at one site. The Avatar Therapy System has been registered as a class 1 medical device by Avatar Therapy Ltd.

### Patient and public involvement

Patient and public involvement (PPI) in which experts by experience supported the study, had a major role at all stages of the AVATAR2 trial, including design, recruitment of staff and participants, analysis and dissemination through supporting the development of accessible plain English summaries and visual representations. An active and creative group of people was established, including members from different backgrounds, with lived experience of mental health conditions and recovery, including carers. In total, the AVATAR2 PPI group included over 20 members across the four sites, with at least four PPI consultants at each site. The local groups met approximately every 2 months for the duration of the trial, with specific activities planned between meetings. There was also coordination of PPI input from sites to the AVATAR2 whole-team events, which took place approximately every 6 months. Group members were reimbursed for their time at a rate of GBP20 per hour; travel expenses were covered for attendance at meetings. Individual members contributed to a wide range of activities: planning events, supporting recruitment, reviewing documents, joining interview and recruitment panels, training research assistants, feeding back on content for the website, reviewing the results and their importance and interpretation, involvement in other public-facing work and attending wider team meetings. Each member was buddied with a named research worker who facilitated flexible and tailored involvement. Personal development plans were a helpful tool to support learning and development within the role. In keeping with principles of open and collaborative involvement, the activities of the PPI group extended beyond those specified within the trial protocol. For example, a creative space was identified as important during PPI meetings and a creative writing workshop emerged organically over time. While independent from the core deliverables of the trial, this regular creative workshop became highly valued and impactful across all aspects of the project (further details of this work will be the focus of a separate publication).

A formal facilitated series of meetings was held with our PPI consultants concerning the outcomes they considered important and reflections on the results. The outcomes considered important are shown in Supplementary Table 6. These are set alongside the most relevant trial outcome measures and whether significant effects were found.

### Concomitant care

Throughout the post-randomization period, participants in all three arms continued with their usual care (TAU). TAU was delivered according to UK national and local service guidelines, typically involving antipsychotic medication, contact with a mental health worker and outpatient psychiatric appointments. Participation did not alter pharmacological or psychosocial treatment decisions. As expected, the TAU-alone arm showed higher levels of other psychological interventions (further data are provided in Supplementary Table 10); 326 (94%) participants were prescribed antipsychotic medication of whom a quarter were prescribed clozapine. Dosages were converted to chlorpromazine equivalents and were broadly comparable between the three arms of the study (Supplementary Table 11).

### Assessment procedures

Assessments were conducted at 0 weeks (baseline) before randomization, 16 weeks (after baseline; follow-up after treatment) and 28 weeks (after baseline; follow-up after 28 weeks). Blinded assessors conducted recruitment and consent procedures and assessments remotely, or at clinics or the participants’ homes. Unblinded site coordinators reviewed electronic clinical notes for the period of participation to collect health economic data.

Assessors were trained to administer the assessment battery by the lead trial coordinator, completed practice assessments and were observed by site coordinators before working independently. Scoring fidelity meetings for the PSYRATS-AH were conducted repeatedly and all assessors attended weekly supervision with coordinators to maintain scoring accuracy and consistency.

Participants were invited to provide additional consent to take part in the ESM assessments at their initial consent into the trial. If they provided consent, they were invited to complete the assessments at every time point (baseline, 16 and 28 weeks). The ESM assessment week consisted of ten questionnaires a day for 6 days and was delivered through the m-Path smartphone application (https://m-path.io/landing/). This provides a self-report of mental state in the flow of daily life. Participants could use their own phones or borrow one from the study team to complete the study. The items contributing to the anxiety score were as follows: right now, I feel: relaxed: 1 not at all, 7 very much so; safe: 1 not at all, 7 very much so; stressed: 1 not at all, 7 very much so; wound up: 1 not at all, 7 very much so; scared: 1 not at all, 7 very much so.

Participants in all three trial arms were compensated GBP20, with an additional GBP15 for the experience sampling assessment, at each time point.

### Study hypotheses

The study investigated the following hypotheses: (1) AV-BRF will be more effective in reducing voice-related distress, total voice severity and voice frequency than TAU after treatment (16 weeks) and at the follow-up (28 weeks); (2) AV-EXT will be more effective in reducing voice-related distress, total voice severity and voice frequency than TAU after treatment (16 weeks) and at the follow-up (28 weeks); (3) greater baseline complexity of voice characterization will moderate the treatment effects of AV-BRF and AV-EXT compared to TAU. Other clinical characteristics will be explored as potential moderators. The following additional study hypotheses will be reported in subsequent publications: (1) AV-EXT will reduce perceived omnipotence and malevolence compared to TAU and these improvements will mediate change in the primary outcome; (2) in both AV-BRF and AV-EXT, the treatment effects on the primary outcome will be mediated by anxiety reduction, as measured by the ESM in daily life; (3) AV-BRF and AV-EXT will both have favorable incremental cost-effectiveness ratios compared to routine care.

We prespecified the interpretation of our results as follows: for each comparison of AVATAR-BRF versus TAU, and AVATAR-EXT versus TAU, if the estimated between-group difference at 16 weeks is statistically significant, we will conclude that there is a treatment effect on the outcome at the end of the intervention period. This will constitute partial support for our hypothesis; if the estimated between-group difference at 28 weeks is statistically significant, we will conclude that there is a treatment effect on the outcome at the follow-up. If there is a statistically significant between-group difference at 28 weeks but not at the earlier 16-week time point, this will constitute partial support for our hypothesis; if there is a statistically significant between-group difference at both time points, we will conclude that the treatment effect is sustained and this will constitute full support for our hypothesis; for the primary outcome of PSYRATS voice-related distress, we will assess the magnitude of the between-group difference against the plausible effect sizes in the sample size calculations.

### Outcomes

The prespecified primary outcome for the study was reduction in distress associated with voices at end of treatment (16 week) and at the follow-up (28 weeks), as measured by the distress dimension of the PSYRATS-AH (five items, distress (two items), negative content (two items) and control^13^. The PSYRATS-AH is a dimensional, semistructured, assessor-rated clinical interview assessing AHs, consisting of 11 items, each item scored from zero (voices not present) to four^26^. Voice-related distress was selected as the primary outcome because it is the central target of the therapy approach and valued as an outcome by experts by experience (Supplementary Table 9).

Key secondary outcomes, as specified in the primary hypotheses, were reductions in the voice frequency scale score (three items: frequency, duration and disruption) and the total severity score (all 11 items) on the PSYRATS-AH Scale at 16 and 28 weeks.

Other secondary outcomes were a mix of assessor-rated and self-reported measures, with effects estimated at 16 and 28 weeks. These included distressing beliefs (PSYRATS-Delusions^26^), well-being (WEMWBS^28^), psychological recovery (CHOICE^29^), fearful attachment (Relationships Questionnaire item^30^), VAAS^31^, measuring acceptance-based attitudes and actions in relation to voice-hearing experiences, mood (DASS^32^ and BDI^33^), anxiety in daily life (using the ESM), voice power (VPDS item^34^) and BAVQ (omnipotence, malevolence and benevolence, total, BAVQ-R^35^), and trauma-related symptoms (ITQ^36^) (16 weeks only).

The clinical characteristics of participants were further assessed at baseline with the Clinical Assessment Interview for Negative Symptoms (CAINS^37^) and Scale for Assessment of Positive Symptoms^38^ (further details of all measures are provided in Supplementary Table 12).

### Safety

All AEs and SAEs were recorded according to the trial standard operating procedure for AEs, following CONSORT guidance, with the extension for social and psychological interventions, and the extension for reporting of harms. The chief investigator reviewed all reports and notified the independent DMEC Chair of any SAEs as they occurred. The DMEC Chair was responsible for reviewing all SAEs and determining the relatedness, if any, of SAEs to the trial procedures (rating as yes, no, possibly related). AEs were recorded for the duration of each participant’s involvement in the trial, from the date on which they signed the consent form until the date of the final assessment or contact with the trial team if they withdrew before their final assessment. Monitoring was conducted by therapists and research assistants, supervised by trial coordinators throughout their contact with participants. After the conclusion of the final assessment for each participant, the trial coordinator reviewed the electronic clinical notes and logged any AEs during their participation in the trial.

All AEs were discussed weekly in trial coordinator meetings and monthly at clinical trial management committee meetings to ensure accurate and consistent monitoring across sites. Where an event was determined to be serious by the site trial coordinator and principal investigator, this form was sent to the DMEC Chair for further review and to determine the rating of the relatedness of the event to any trial procedure.

The criteria for determining whether an incident should be considered a serious or nonserious AE are shown below and were included within a standard operating procedure for AE reporting, followed by all staff during the trial. An AE was defined as: any untoward medical occurrence, unintended disease or injury, or untoward clinical signs in participants that lead to significant increased distress and interference with daily life such that intervention from the clinical team was required.

This included AEs related to both intervention arms of the AVATAR2 trial and to the TAU group, and to all research procedures involved. It was anticipated that participants might experience some distress in relation to the assessment measures or treatment processes. If this distress was managed by the trial team and did not require additional support from clinical services, then this was not classified as an AE.

An AE was defined as serious (that is, an SAE) by the ISO 14155:2011 guidelines for medical device trials if it: resulted in death OR was a life-threatening illness or injury OR required (voluntary or involuntary) hospitalization or prolongation of existing hospitalization OR resulted in persistent or significant disability or incapacity OR medical or surgical intervention was required to prevent any of the above OR led to fetal distress, fetal death or consisted of a congenital anomaly or birth defect OR was otherwise considered medically significant by the investigator.

Life-threatening in the definition of an SAE refers to an event in which the individual was at risk of death at the time of the event; it does not refer to an event that might hypothetically have caused death if it were more severe. Events that are not immediately life-threatening or do not result in death or hospitalization but may jeopardize the individual or may require intervention to prevent one or the other outcomes listed, should be considered serious.

A planned hospitalization for a preexisting condition, without a serious deterioration in health, is not considered an SAE.

### Statistical analysis and sample size calculations

We powered the study to detect plausible effect sizes based on our previous AVATAR therapy trial^8^. There we found a clinically meaningful reduction in PSYRATS-AH distress of 4.8 points, with an effect size of approximately *d* = 0.8, but we reduced this for the current trial to take into consideration the increase in number of centers, the follow-up comparison (not only the end of treatment) and a more pragmatic trial design. We are accounting for two formal comparisons: AV-EXT versus TAU—plausible effect size = 0.6; and AV-BRF versus TAU—plausible effect size = 0.5. The study was powered for an overall treatment effect at a 5% significance level, accounting for two multiple group comparisons in which the tests are correlated because of shared control data (at *r* = 0.5), giving an alpha level for each group-specific test of 0.035. Accordingly, a sample size of 92 per group or 276 in total in the analysis dataset had 90% power to detect a minimum clinically significant difference (effect size) of 0.5 standard deviations. We sought to recruit 345 participants at baseline (87 per site), with *n* = 115 per treatment arm, allowing for a conservative attrition rate of 20%.

We report the findings in line with the most recent relevant CONSORT guidelines, the 2018 extension for reporting social and psychological intervention trials^39^. No interim analysis was performed. All analyses were conducted in Stata v.18.1 (ref. ^40^). To visualize the data, R v.4.33 (ref. ^41^) and ggplot2 v.3.5.0 (ref. ^42^) were used. The senior statistician (R.E.) was unblinded only after completion of the initial analyses and presentation of these results to our external advisory committees. The junior statistician (H.J.) was unblinded during the study after preparing the first closed DMEC report; the statistical analysis was performed unblinded owing to the need to account for therapist effects in the AVATAR arms.

The primary estimand is the treatment policy estimand. The primary analyses were carried out using the ITT sample: participants were analyzed in the group they were randomized to; available data from all participants are included, including those who did not complete treatment.

The primary analyses of the hypotheses of between-group differences in the AV-EXT versus TAU and AV-BRF versus TAU in voice distress as measured using the PSYRATS-AH distress score were analyzed using a mixed-effects (random) model at all post-randomization time points (weeks 16 and 28). Fixed effects were the center, baseline assessment for the outcome under investigation, voice characterization (low or /high), treatment, time (categorical, 16 or 28 weeks) and time × treatment interactions. Marginal treatment effects were estimated for the outcomes at each time point and reported separately as adjusted mean differences in scores between the randomized groups with 96.5% CIs and two-sided *P* values.

To account for the partial nested design, we included a random intercept for therapist in the treatment arms only, with the participants in the TAU arm considered as being in individual clusters of size one. The same therapists delivered both AV-EXT and AV-BRF. Participants in the intervention arms who did not attend any sessions with a therapist were nominally allocated to a single therapist ID for ITT purposes. Participant was included as a random intercept nested within therapist to account for repeated measures of outcomes.

For the continuous secondary outcomes we followed the same model as the primary analysis: linear mixed models, including the outcome measures at all post-randomization time points, with a time by treatment interaction to allow the estimation of the between-arm difference at each time point.

All statistical models were estimated using maximum likelihood estimation, which allows for missing outcome data under the missing at random assumption. In addition, we report estimates for Cohen’s *d* effect sizes at 16 and 28 weeks as the adjusted mean difference of the outcome divided by the sample s.d. of the outcome at baseline. CIs for Cohen’s *d* were calculated by dividing the 96.5% confidence limits by the sample s.d. of the outcome at baseline. These are displayed in a forest plot with the primary outcome at the top, followed by key secondary and other outcomes, with a separate plot for each time point.

The moderation analysis investigated how a prespecified set of putative baseline moderators affected the efficacy of the treatment interventions (TAU, AV-BRF, AV-EXT) in reducing the distress associated with AHs over time at 16 and 28 weeks. Specifically, the interaction effects between treatment groups, time and moderator were analyzed to understand the differential influence of moderator on the treatment effects between TAU versus AV-EXT and TAU versus AV-BRF. We investigated the specific hypothesis that greater baseline complexity of voice characterization would moderate the treatment effects of AVATAR-BRF and AVATAR-EXT compared to TAU. The following measures of baseline clinical and cognitive characteristics were also considered as potential moderators of treatment effects: PSYRATS-AH-Distress, trauma-related symptoms: PTSD, DSO, negative symptoms (CAINS for motivation and pleasure, and CAINS for expressiveness), duration of mental health services (early versus not early), duration of hearing voices, age voices started and attachment (Relationship Questionnaire). We also examined demographic variables as moderators: age, gender and self-defined ethnicity.

For a continuous moderator, the difference in treatment effect between the unit levels of the moderator can be interpreted as the difference in the estimated treatment effect between a participant with a moderator value at baseline of *a* + 1 and a participant with a moderator value at baseline of *a*. For a binary moderator (for example, low versus high voice characterization), the difference in treatment effect can be interpreted as the difference in the estimated treatment effect between participants with low and those with high voice characterization.

CACE compares the average outcome between those in the AV-EXT and AV-BRF groups who meet the definition of receiving a minimal treatment dose and the latent subgroup of people in the control group who would have received this dose had they been randomized to the respective intervention group (that is, this is a hidden counterfactual). For each outcome, we would expect to see an increased effect estimate for CACE relative to the ITT effect because we are systematically excluding people who do not receive the treatment dose of AV-EXT or AV-BRF. However, the statistical significance does not necessarily increase because the instrumental variable method used to calculate the CACE estimates produces larger standard errors as a consequence of accounting for the selection effects between those who receive and those who do not receive a treatment dose in the intervention arm. As such, this is best seen as a bias correction that answers the question ‘what is the effect of offering AVATAR compared to TAU in those who would receive a treatment dose of AVATAR if offered?’

We estimated the CACE for each comparison of AV-EXT versus TAU, and AV-BRF versus TAU, separately; this means that we excluded from the model the AVATAR intervention group not being used in the comparison. We used an instrumental variable method with two-stage least squares estimation and robust standard errors to account for clustering by therapist. We estimated the effect for each time point using separate analyses. Randomization was used as the instrument; receipt of a minimal treatment dose was the endogenous (treatment received) variable. We included the same set of covariates as the primary analysis models in both stage regressions.

Further details of the statistical methods, including the treatment of missing data, are provided in the statistical analysis plan (published in the ISRCTN registry with the identifier ISRCTN55682735). A post hoc sensitivity analysis for missing data in the primary outcome is provided in Supplementary Table 13 and Supplementary Fig. 1).

### Reporting summary

Further information on research design is available in the Nature Portfolio Reporting Summary linked to this article.

## Online content

Any methods, additional references, Nature Portfolio reporting summaries, source data, extended data, supplementary information, acknowledgements, peer review information; details of author contributions and competing interests; and statements of data and code availability are available at 10.1038/s41591-024-03252-8.

## Supplementary information

Supplementary Information 1 Therapy completion and delivery 2. 2 Serious adverse events and adverse events further data 3. 3 Patient and public involvement—further details 5. 4 Concomitant care 5. 5 Outcome measures 6. 6 Post hoc sensitivity analysis for missing data in primary outcome 7.

Reporting Summary

## Figures and Tables

**Extended Data Table 1:**
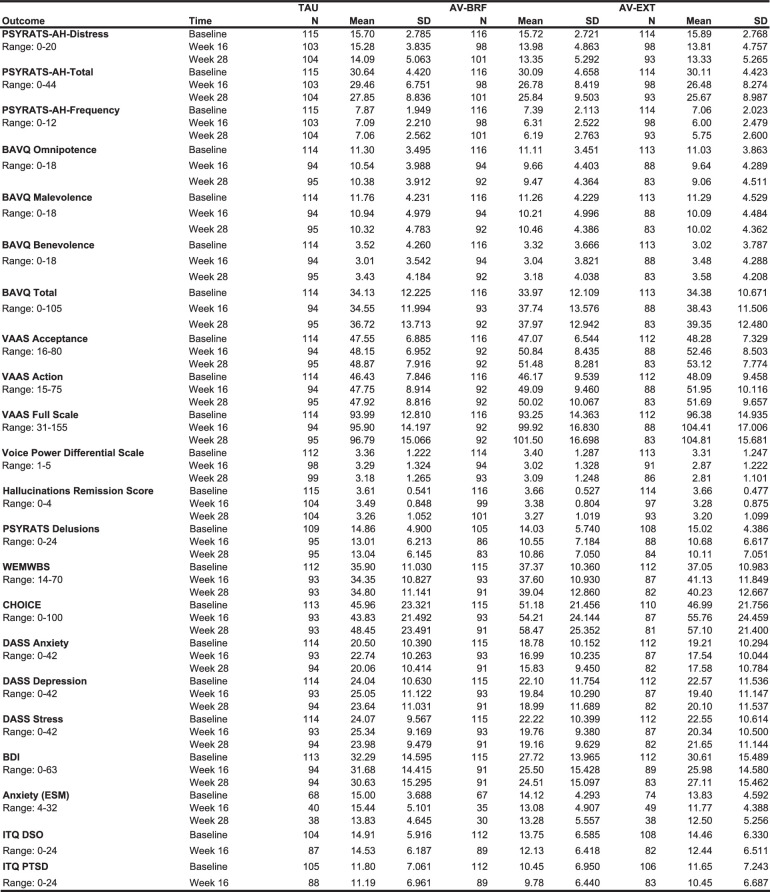
Descriptive statistics for primary and other secondary voice-related outcomes across three treatment groups at each time point

AH, auditory hallucinations; AV-BRF, Brief AVATAR therapy; AV-EXT, Extended AVATAR Therapy; BAVQ, Beliefs About Voices Questionnaire; N: Number, PSYRATS: Psychotic Symptom Rating Scales; TAU, treatment as usual; VAAS, Voices Acceptance And Action Scale. Time points: 16, 16 weeks after baseline (after-treatment follow-up); 28, 28 weeks after the baseline follow-up.

**Extended Data Table 2:**
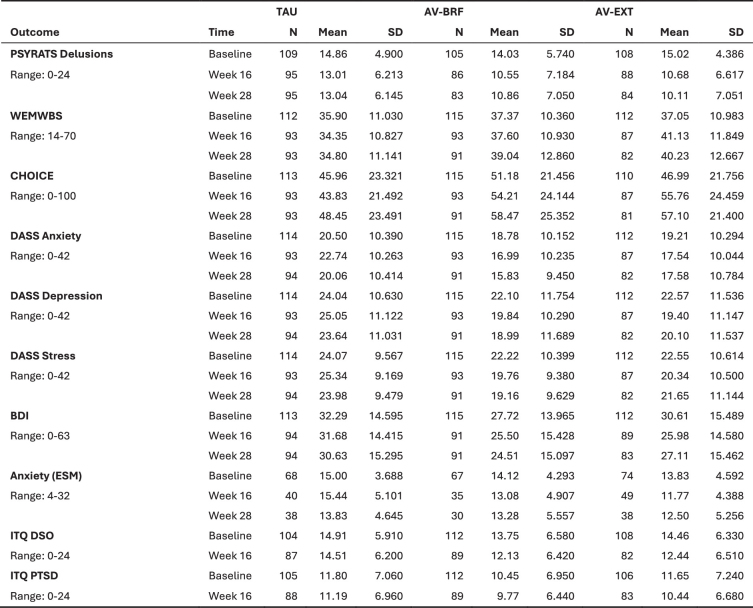
Descriptive statistics for other secondary outcomes across three treatment groups at each time point

AV-BRF, Brief AVATAR therapy; AV-EXT, Extended AVATAR Therapy; BDI, Beck Depression Inventory; CHOICE, CHoice of Outcome In Cbt for PsychosEs; DASS, Depression Anxiety Stress Scales; DSO, Disturbances in self-organization; ESM, Experience Sampling Method; ITQ, International Trauma Questionnaire; PTSD, Post-Traumatic Stress Disorder; TAU, treatment as usual; WEMWBS, Warwick-Edinburgh Mental Well-being Scale. Time points: 16, 16 weeks after baseline (after-treatment follow-up); 28, 28 weeks after the baseline follow-up.

**Extended Data Table 3:**
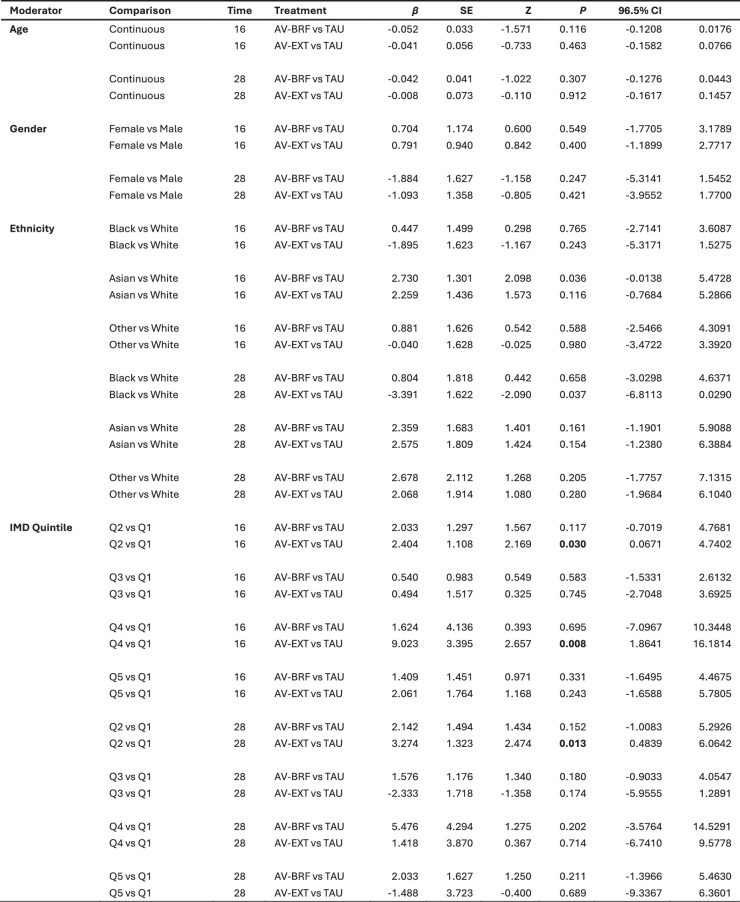
Moderation analysis for demographic variables: estimated margins of interaction effect between moderator variable and treatment group, including 96.5% confidence intervals

AV-BRF, Brief AVATAR therapy; AV-EXT, Extended AVATAR therapy; IMD, Index of Multiple Deprivation; TAU, treatment as usual. Time points: 16, 16 weeks after baseline (after-treatment follow-up); 28, 28 weeks after the baseline follow-up. Mixed-effects regression models were used for the moderation analysis, with robust standard error estimation. Two-sided tests were applied, with adjustments for multiple comparisons using a significance level of 0.035 and 96.5% confidence intervals reported. The *β* coefficients represent the difference in treatment effect estimates attributable to changes between the levels of the moderator, as specified in the ‘Comparison’ column, for each treatment condition shown in the ‘Treatment’ column. Bold denotes *P* ≤ 0.035.

**Extended Data Table 4:**
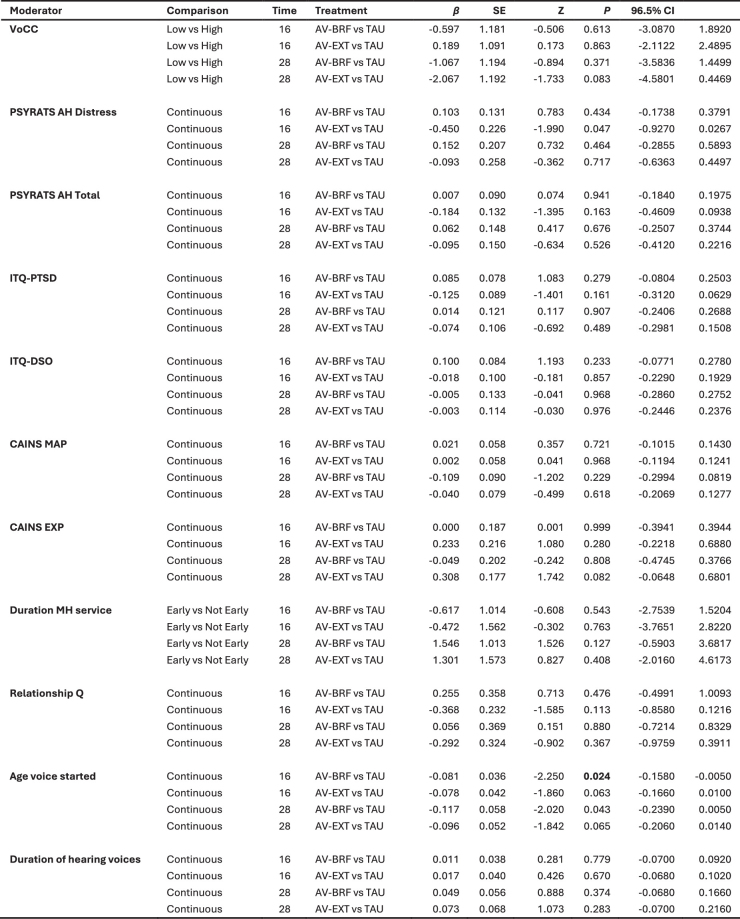
Moderation analysis for clinical variables: estimated margins of interaction effect between moderator variable and treatment group, including 96.5% confidence intervals

AH, auditory hallucinations; AV-BRF, Brief AVATAR therapy; AV-EXT, Extended AVATAR therapy; DSO, Disturbances in self-organization; ITQ, International Trauma Questionnaire; MH, mental health; PSYRATS, Psychotic Symptom Rating Scales; PTSD, Post-Traumatic Stress Disorder; TAU, treatment as usual; VoCC, Voice Characterization Checklist. Time points: 16, 16 weeks after baseline (after-treatment follow-up); 28, 28 weeks after the baseline follow-up. Mixed-effects regression models were used for the analysis, with robust standard error estimation. Two-sided tests were applied, with adjustments for multiple comparisons using a significance level of 0.035 and 96.5% confidence intervals reported. The *β* coefficients represent the difference in treatment effect estimates attributable to changes between the levels of the moderator, as specified in the ‘Comparison’ column, for each treatment condition shown in the ‘Treatment’ column. Bold denotes *P* ≤ 0.035.

**Extended Data Table 5:**
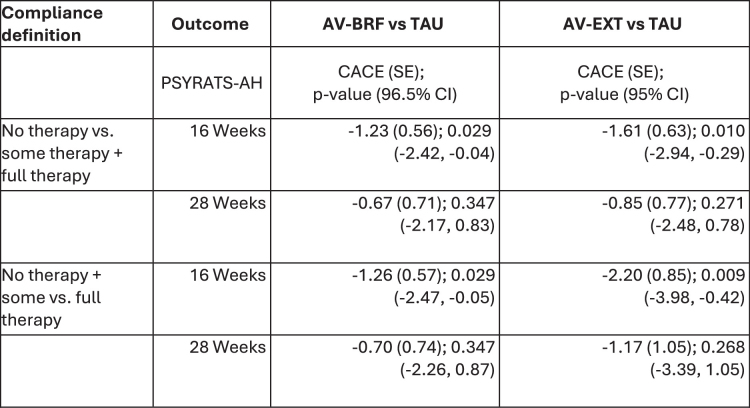
Compliance-adjusted analysis

AH, auditory hallucinations; AV-BRF, Brief AVATAR therapy; AV-EXT, Extended AVATAR Therapy; CACE, Compliance Adjusted Causal Effects; PSYRATS, Psychotic Symptom Rating Scales; TAU, treatment as usual.

## Data Availability

Open access information on the AVATAR2 trial, such as the trial protocol and statistical analysis plan, including the example analysis code, has been published in the ISRCTN registry with the identifier ISRCTN55682735; the final trial protocol (v.1.2) was also published in *T**rials*^11^. Individual participant data have been deposited in the King’s Open Research Data System, but access is restricted due to privacy reasons and general data protection regulations, and can only be accessed after review. Data will be made accessible after the publication of this paper. A request can be made by academic or clinical researchers to research.data@kcl.ac.uk for the purpose of conducting noncommercial, ethically approved research. The research data team will review the request against the conditions set out in the Data Access Agreement. An initial response to requests will be formulated within a month. A Data Access Agreement will be drawn up before data can be shared.

## Code availability

In accordance with the data availability protocol, the corresponding statistical code will be provided as part of the Data Access Agreement.
