# Author Correction: Digital AVATAR therapy for distressing voices in psychosis: the phase 2/3 AVATAR2 trial

**DOI:** 10.1038/s41591-026-04540-1

**Published:** 2026-06-30

**Authors:** Philippa A. Garety, Clementine J. Edwards, Hassan Jafari, Richard Emsley, Mark Huckvale, Mar Rus-Calafell, Miriam Fornells-Ambrojo, Andrew Gumley, Gillian Haddock, Sandra Bucci, Hamish J. McLeod, Jeffrey McDonnell, Moya Clancy, Michael Fitzsimmons, Hannah Ball, Alice Montague, Nikos Xanidis, Amy Hardy, Thomas K. J. Craig, Thomas Ward

**Affiliations:** 1 Department of Psychology, Institute of Psychiatry, Psychology & Neuroscience, King’s College London, London, UK; 2 South London & Maudsley NHS Foundation Trust, London, UK; 3 Department of Biostatistics and Health Informatics, Institute of Psychiatry, Psychology and Neuroscience, King’s College London, London, UK; 4 University College London, London, UK; 5 Mental Health Research and Treatment Center, Faculty of Psychology, Ruhr-Universität Bochum, Bochum, Germany; 6 Research Department of Clinical, Educational and Health Psychology, University College London, London, UK; 7 North East London NHS Foundation Trust, London, UK; 8 School of Health and Wellbeing, University of Glasgow, Glasgow, UK; 9 NHS Greater Glasgow & Clyde, Glasgow, UK; 10 Division of Psychology and Mental Health, School of Health Sciences, University of Manchester and the Manchester Academic Health Sciences Centre, Manchester, UK; 11 Greater Manchester Mental Health NHS Foundation Trust and the Manchester Academic Health Sciences Centre, Manchester, UK; 12 Department of Health Service and Population Research, Institute of Psychiatry, Psychology & Neuroscience, King’s College London, London, UK

**Keywords:** Outcomes research, Randomized controlled trials

Correction to: *Nature Medicine* 10.1038/s41591-024-03252-8, published online 28 October 2024.

In the version of this article initially published, there were errors in Table 2 and Extended Data Table 1: item scoring for the BAVQ Omnipotence and Malevolence subscales was applied in the incorrect direction, and there was a minor coding error in the algorithm used to prorate missing items when computing the ITQ-DSO and ITQ-PTSD subscale scores. For the inversion error, none of the comparisons for the subscales were statistically significant in the submitted version, and none are in the corrected version, with no impact on any inferential conclusion. For the coding error, corrections are confined to the third and fourth decimal places of the effect estimates in Table 2, and there were only marginal differences in the means and standard deviations in Extended Table 1. The significance classification for all *P* values is unaffected. For comparison, the original and revised tables are available as Supplementary Information accompanying this amendment. The tables have been updated in the HTML and PDF versions of the article.

## Supplementary information

Original and revised Table 2, Extended Data Table 1

