# Supplementary material for: Digital AVATAR therapy for distressing voices in psychosis: the phase 2/3 AVATAR2 trial
Source: Nat Med. 2024 Oct 28;30(12):3658–68. doi: 10.1038/s41591-024-03252-8 (PMC11645260; doi:10.1038/s41591-024-03252-8)
Supplement: Supplementary file 1 — 1 Therapy completion and delivery 2. 2 Serious adverse events and adverse events further data 3. 3 Patient and public involvement—further details 5. 4 Concomitant care 5. 5 Outcome measures 6. 6 Post hoc sensitivity analysis for missing data in primary outcome 7. [file 41591_2024_3252_MOESM1_ESM.pdf]

nature medicine

Supplementary information https://doi.org/10.1038/s41591-024-03252-8

Digital AVATAR therapy for distressing voices in psychosis: the phase 2/3 AVATAR2 trial

In the format provided by the authors and unedited

Supplementary Materials (AVATAR2 trial)

Contents

1. Therapy completion and delivery 2
2. Serious Adverse Events (SAEs) and Adverse Events (AEs) Further Data 3
3. Patient and Public Involvement (PPI) – further details 5
4. Concomitant care 5
5. Outcome measures 6
6. Post-hoc sensitivity analysis for missing data in primary outcome 7

1. Therapy completion and delivery

	Intervention Group and Gender					
	AV-BRF			AV-EXT		
	**Male**	**Female**	**Other**	**Male**	**Female**	**Other**
N	72 (62.1%)	43 (37.1%)	1 (0.9%)	71 (62.3%)	42 (36.8%)	1 (0.9%)
**Therapy completion**						
No therapy	11 (15.3%)	6 (14.0%)	0 (0.0%)	7 (9.9%)	4 (9.5%)	0 (0.0%)
Partial therapy	2 (2.8%)	2 (4.7%)	0 (0.0%)	21 (29.6%)	15 (35.7%)	1 (100.0%)
Completed therapy	59 (81.9%)	35 (81.4%)	1 (100.0%)	43 (60.6%)	23 (54.8%)	0 (0.0%)

Table s1: Therapy completion by arm and gender

As shown in Table s1 rates of therapy completion are comparable across gender for both AV-BRF and AV-EXT.

	Intervention Group and Ethnicity							
	AV-BRF				AV-EXT			
	**White**	**Black or mixed Black**	**South Asian or mixed South Asian**	**Other**	**White**	**Black or mixed Black**	**South Asian or mixed South Asian**	**Other**
N	63 (54.3%)	19 (16.4%)	9 (7.8%)	25 (21.6%)	71 (62.3%)	19 (16.7%)	6 (5.3%)	18 (15.8%)
**Therapy completion**								
No therapy	12 (19.0%)	1 (5.3%)	0 (0.0%)	4 (16.0%)	7 (9.9%)	2 (10.5%)	1 (16.7%)	1 (5.6%)
Partial therapy	1 (1.6%)	1 (5.3%)	2 (22.2%)	0 (0.0%)	25 (35.2%)	3 (15.8%)	4 (66.7%)	5 (27.8%)
Completed therapy	50 (79.4%)	17 (89.5%)	7 (77.8%)	21 (84.0%)	39 (54.9%)	14 (73.7%)	1 (16.7%)	12 (66.7%)

Table s2: Therapy completion by arm and ethnicity

As shown in Table s2, for AV-BRF therapy completion rates are comparable across all groups, with the “Black or mixed Black” group showing the highest rate numerically. For AV-EXT the “Black or mixed Black” group again shows the highest percentage for completed therapy. The “South Asian or mixed South Asian” is lower than the other categories. However, this may be an artefact of the small number in this category (n=6).

Therapy Delivery mode

Delivery mode	KCL	UCL	Manchester	Glasgow	Total (n)	Total (%)
**Face-to-face**	47	50	54	49	200	87.0
**Fully remote**	11	8	1	1	21	9.1
**Combination (F2F/remote)**	1	1	4	3	9	3.9
**% per site remote/combination**	20.3	15.3	8.5	7.5		

Table s3: Delivery mode across the four trial sites

		Number (n); total n=230	%
**AVATAR-EXT**	**F2F**	98	86.0
	**Remote**	14	12.3
	**Combination**	2	1.8
**AVATAR-BRF**	**F2F**	102	87.9
	**Remote**	7	6.0
	**Combination**	7	6.0

Table s4: Data on delivery mode across AV-EXT and AV-BRF trial arms

2. Serious Adverse Events (SAEs) and Adverse Events (AEs) Further Data

<table>
<tr><th></th><th>Non-trial related</th><th colspan="2">Therapy related</th><th colspan="2">Assessment related</th><th colspan="2">Device related</th></tr>
<tr><td>Non-serious</td><td>Adverse Event (AE)</td><td colspan="2">Therapy Related Adverse Event (TRAE)</td><td colspan="2">Assessment Related Adverse Event (ARAE)</td><td colspan="2">Adverse Device Effect (ADE)</td></tr>
<tr><td rowspan="2">Serious</td><td rowspan="2">Serious Adverse Event (SAE)</td><td colspan="2">Serious Therapy Related Adverse Event (STRAE)</td><td colspan="2">Serious Assessment Related Adverse Event (SARAE)</td><td colspan="2">Serious Adverse Device Effect (SADE)</td></tr>
<tr><td>Anticipated Serious Therapy Related Adverse Event (ASTRAE)</td><td>Unanticipated Serious Therapy Related Adverse Event (USTRAE)</td><td>Anticipated Serious Assessment Related Adverse Event (ASARAE)</td><td>Unanticipated Serious Assessment Related Adverse Event (USARAE)</td><td>Anticipated Serious Adverse Device Effect (ASADE)</td><td>Unanticipated Serious Adverse Device Effect (USADE)</td></tr>
</table>

Table s5: Definitions relating to the monitoring and reporting of serious and non-serious AEs.

	**TAU N=67**	**AV-BRF N=59**	**AV-EXT N=113**
AE	53 (79.1%)	45 (76.3%)	83 (73.5%)
SAE: Death	-	-	2 (1.8%)

SAE: Incidents which acutely jeopardise the health or psychological wellbeing	13 (19.4%)	12 (20.3%)	19 (16.8%)
SAE: Resulting in injury requiring immediate medical attention	1 (1.5%)	2 (3.4%)	9 (8.0%)

Table s6: All adverse events (SAEs and AEs), by trial arm

	Trial arm		
**Relatedness**	**TAU**	**AV-BRF**	**AV-EXT**
	Number of P, E (%) of SAEs		
**Therapy related**			
No	13, 14 (100.0%)	13, 13 (92.9%)	19, 25 (83.3%)
Possibly	0 (0.0%)	1, 1 (7.1%)	4, 5 (16.7%)
**Device related**			
No	13, 14 (100.0%)	14, 14 (100.0%)	23, 30 (100.0%)
**Assessment related**			
No	13, 14 (100.0%)	14, 14 (100.0%)	23, 30 (100.0%)

Table s7: Data on relatedness (rated by the independent DMEC chair) *of SAEs by arm (Numbers: P, E (%): Participant, Events (% of events))*

	Trial arm			
	**TAU P, E (%)**	**AV-BRF P, E (%)**	**AV-EXT P, E (%)**	**Total P, E (%)**
N	39, 53 (29.3%)	35, 45 (24.9%)	56, 83 (45.9%)	130, 181 (100.0%)
**Adverse event type**				
Distress associated with completion of assessment measures	1, 1 (1.9%)	3, 3 (6.7%)	2, 2 (2.4%)	6, 6 (3.3%)
Significant distress during the AVATAR therapy	0 (0.0%)	2, 2 (4.4%)	6, 6 (7.2%)	8, 8 (4.4%)
Referral to crisis team	3, 4 (7.5%)	3, 3 (6.7%)	0, 0 (0.0%)	6, 7 (3.9%)
Violent incident necessitating police involvement (victim)	0 (0.0%)	0 (0.0%)	1, 1 (1.2%)	1, 1 (0.6%)
Violent incident necessitating police involvement (accused)	1, 1 (1.9%)	0 (0.0%)	1, 1 (1.2%)	2, 2 (1.1%)
Deliberate self-harm	2, 2 (3.8%)	3, 4 (8.9%)	1, 1 (1.2%)	6, 7 (3.9%)
Other psychological health event	26, 36 (67.9%)	21, 30 (66.7%)	34, 59 (71.1%)	81, 125 (69.1%)
Other physical health event	6, 9 (17.0%)	3, 3 (6.7%)	11, 13 (15.7%)	20, 25 (13.8%)

Table s8: Data on AEs by arm (Numbers: P, E (%): Participant, Events (% of events))

3. Patient and Public Involvement (PPI) – further details

Priorities for AVATAR2 Experts by Experience	Associated Outcome	AV-BRF (16)	AV-BRF (28)	AV-EXT (16)	AV-EXT (28)
"To get rid of the voices completely"	PSYRATS (FRQ)	X	X	✓	✓
"To help the voices become less nasty, less harmful"	PSYRATS (DIS)	✓	X	✓	X
"Living a life less affected by voice hearing" (wellbeing)	WEMWBS	✓	X	✓	✓
"Getting control of your life"	Voice Action (VAAS)	✓	✓	✓	✓
Being at peace with voice hearing	Voice acceptance (VAAS)	✓	✓	✓	✓

Table s9: PPI priority outcomes and whether significant effects at each time point (indicated by ticks)

4. Concomitant care

a) Psychological/ psychosocial interventions

Trial Arm	Format	Participants N	Interventions N (%)	Was the focus on distressing voices?	N (%)
**AV-EXT**	Individual	16	17 (14.7%)		
	Family	2	2 (1.7%)		
	Group	8	8 (6.9%)	Yes	6 (22.2%)
	No interventions	89	89 (76.7%)	No	21 (77.8%)
**AV-BRF**	Individual	18	18 (14.8%)		
	Family	2	2 (1.6%)		
	Group	14	15 (12.3%)	Yes	8 (22.9%)
	No interventions	87	87 (71.3%)	No	27 (77.1%)
**TAU**	Individual	35	38 (27.9%)		
	Family	5	5 (3.7%)		
	Group	15	21 (15.4%)	Yes	22 (34.4%)
	No interventions	72	72 (52.9%)	No	42 (65.6%)

Table s10: Data on psychological interventions (other than AVATAR therapy) by trial arm

b) Antipsychotic Medication

Study Arm	Chlorpromazine equivalent mg/d (mean (sd))
**AV-EXT**	482.3 (343.0)
**AV-BRF**	501.1 (383.3)
**TAU**	451.5 (300.2)

Table s11: Data on chlorpromazine equivalents by arm

5. Outcome measures

Measure	Measure	Time-point*
**Primary Outcome Measure**		
Psychotic Symptom Rating Scales – Auditory Hallucinations (Haddock et al,1999).	The PSYRATS is an interview measure assessing the severity of auditory hallucinations across a range of domains, a higher score indicates higher severity (range is 0-44). The scale includes frequency (range 0-12) and distress (range 0-20) subscales.	Screening, 1, 2, 3
**Secondary Outcome Measures**		
Hallucinations Remission Score	This is a single item assessing the current frequency of voice-hearing experiences, higher score indicates the person has more recently heard a voice, range is 0-4.	1, 2, 3
Beliefs about Voices Revised (BAVQ-R) (Chadwick et al, 2000)	The BAVQ is a self-report measure assessing beliefs people hold about their voice-hearing experiences, subscales measures beliefs about malevolent, omnipotent, or benevolent abilities and intentions – a higher score indicates greater endorsement of these beliefs, range is 0-18. Further subscales also assess emotional and behavioural resistance and engagement with the voices, these subscales have a range of 0-12 except resistance behaviour which is 0-15.	1, 2, 3
Voices acceptance and action scale (VAAS) (Shawyer et al, 2007)	The VAAS is a self-report measure of acceptance-based attitudes and actions in relation to voice-hearing experiences. A higher score indicates that someone is endorsing more acceptance-based attitudes and actions (range is 31-155).	1, 2, 3
The power item from the Voice Power Differential Scale (Birchwood et al., 2000)	This is a self-report single item focused on where the person perceives the power is – with them or the voice. Higher score indicates more power with the voice, and lower scores means more power with the person (range is 0-5.)	1, 2, 3
Beck Depression Inventory-II (Beck et al., 1996)	A detailed self-report measure of depressive symptoms (mental and physical). A higher score indicates more severe symptoms with a range of 0-63.	1, 2, 3

Depression Anxiety and Stress Scales (DASS-21) (Henry & Crawford, 2005)	A self-report measure assessing symptoms of depression, stress and anxiety, each subscale is reported separately, and a higher score indicates worse symptoms, with a range of 0-21.	1,2,3
Warwick-Edinburgh Mental Well-being Scale (Tennant et al., 2007)	This is a self-report measure of mental well-being, a higher score indicates better well-being, range is 0-70.	1, 2, 3
Delusional beliefs subscale of the Psychotic Symptoms Rating Scale (PSYRATS-DEL) (Haddock et al., 1999)	This is an interview measure which assess the severity of delusional beliefs (preoccupation, distress, disruption), a higher score indicates higher severity, range is 0-18)	1, 2, 3
Choice of Outcome in CBT for Psychosis (CHOICE) Short Form (Greenwood et al., 2010)	The CHOICE is. a self-report measure of psychological recovery, co-designed to reflect the priorities of service users. A higher score indicates greater psychological recovery, range is 0-110.	1,2,3
International Trauma Questionnaire (Cloitre et al., 2018)	A self-report measure focused on the core features of post-traumatic stress disorder (PTSD) and complex-PTSD. Scoring algorithms can be applied to ascertain whether a score meets threshold for diagnosis, or to calculate PTSD and Disturbances in Self-Organisation (DSO) clusters (range is 0-24 for each cluster).	1, 2
**Baseline Clinical Characteristics**		
The Voice Characterisation Checklist (Edwards et al, 2023)	Characterisation of voice entity complexity: more or less high characterisation of the voice as an identifiable and characterful entity, based on physical characteristics, identity (e.g. known, name), psychosocial characteristics (e.g. relationship, ascribed intentions and thoughts). A score of 7 or higher was categorised as a more highly characterised voice and the sample was stratified on the basis of this categorisation (more or less highly characterised).	1
Clinical Assessment Interview for Negative Symptoms (CAINS) (Kring et al., 2013)	An interview measure with two subscales, experiential negative symptoms (range is 0-27) and expressive negative symptoms (range is 0-12).	1
Scale for the Assessment of Positive Symptoms (Andreasen, 1984)	An interview measure of positive symptoms, including subscales for hallucinations (0-35), delusions (0-65), bizarre behaviour (0-25) and formal thought disorder (0-45).	1
The Relationships Questionnaire (RQ), Fearful Attachment Item (Bartholomew & Horowitz, 1991).	A self-report item assessing fearful attachment, range is 1-7).	1

Table s12: Detail on all outcome measures and timing of data collection

6. Post-hoc sensitivity analysis for missing data in primary outcome

This is a post-hoc sensitivity analysis conducted at the request of a peer-reviewer that was not pre-specified in our Statistical Analysis Plan.

Follow-up time		Treatment group (N total = 345)			
		TAU	Brief	Extended	Total
**Week 16**	Missed	12	18	16	46
	Available	103	98	98	299
**Week 28**	Missed	11	15	21	47
	Available	104	101	93	298

Table s13. Missing data in primary outcome by randomised group at week 16 and week 28

Table s13 shows the amount of missing primary outcome data by randomised group at each time point. This sensitivity analysis evaluates the impact of missing outcome data on the estimates of the between-group treatment effects on the primary outcome, "Distress associated with voices," using a pattern-mixture model approach. We use the "rctmiss" module in Stata, designed for single time-point regression analysis in two-arm trials. A sensitivity parameter (delta) measures the degree of departure from missing at random by modelling the differences between missing and observed outcomes. A range of delta values from -5 to -5 was applied to explore the impact of missing outcome data on our conclusions. Note that applying a delta range from -5 to 5 in an outcome measure with a range of 0 to 20 is an extreme situation that we do not expect to occur. This approach helps us understand the robustness of our findings under very unlikely scenarios.

Figure s1 shows the results of the sensitivity analysis for the treatment effect estimates for each pairwise comparison at 16 and 28 weeks. The x-axis shows the value of delta ranging from -5 to 5, where 0 corresponds to the primary analysis under a missing at random assumption. A value of -5 is interpreted as the mean outcome of participants with a missing outcome is 5 points lower than those with an observed outcome (i.e. less distress). A value of +5 is interpreted as the mean outcome of participants with a missing outcome is 5 points higher than those with an observed outcome (i.e. more distress). This delta can be applied to the intervention group only (blue line), the TAU group only (green line) or both groups simultaneously (red line).

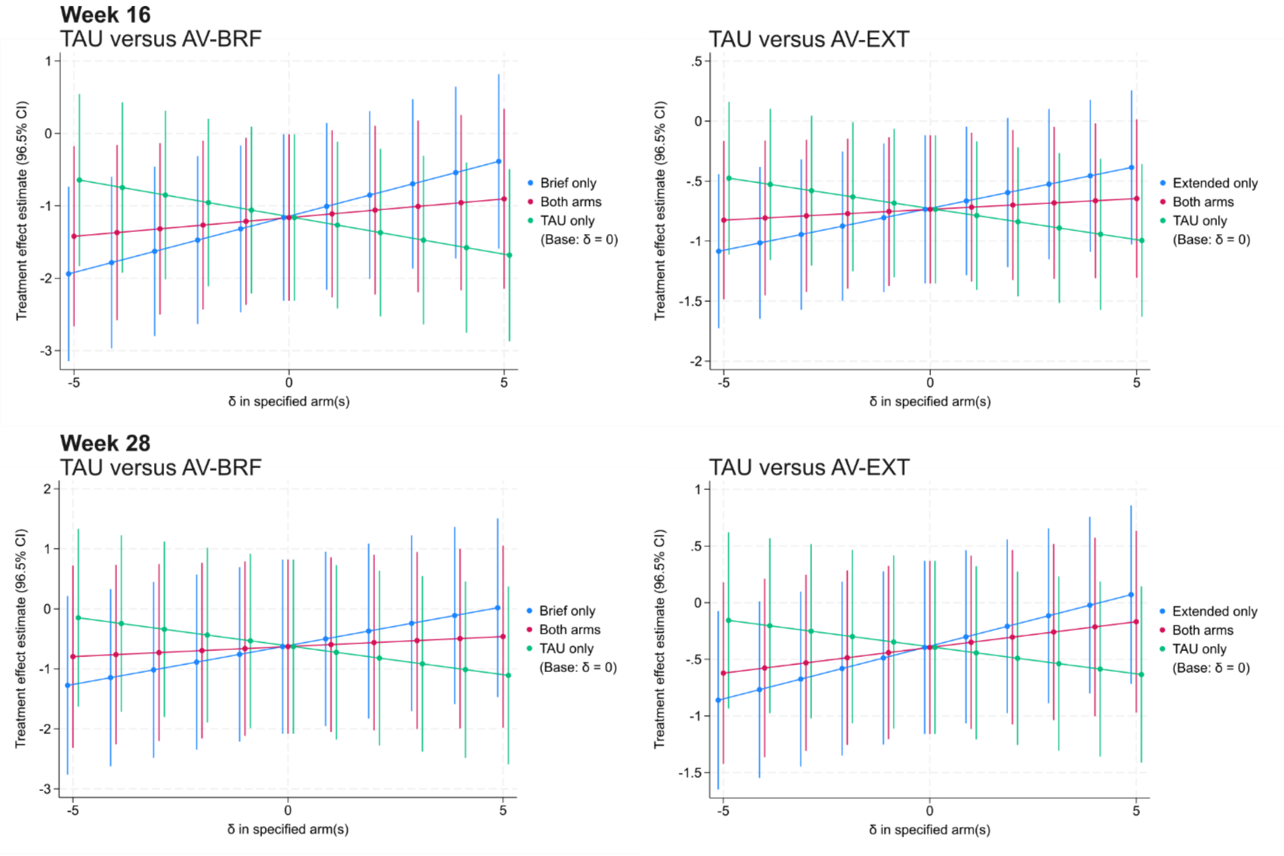

Figure s1: Results of sensitivity analysis for treatment effect estimates with 96.5% confidence intervals at 16 and 28 weeks. The central points represent the estimated between-group difference derived from the sensitivity analysis, with error bars indicating the 96.5% confidence intervals for the difference.

For week 16, the sensitivity analysis demonstrates that the treatment effect estimates are robust to deviations from the missing at random (MAR) assumption for both comparisons. For AV-BRF, the point estimates remain in favour of AV-BRF across all scenarios, though the confidence intervals include 0 (null effect) when the average scores of the missing participants are lower (better) in the control group only, or higher (worse) in the AV-BRF group only. The same pattern is seen for AV-EXT compared to TAU.

For week 28, the pattern of results is similar to week 16 for both comparisons. All point estimates are less than 0 (favour intervention) except the extreme scenario where the missing participants in the intervention group only have 5 points higher mean scores than those observed in that group. The confidence intervals include 0 in more scenarios and this is consistent with the primary analysis in the main paper.

The findings and use of delta values ranging from -5 to 5, which encompasses scenarios with both better and worse distress scores for missing participants, confirms the robustness of our study's results. This reinforces confidence that the observed treatment effects are not substantially biased by the missing data or the missing data modelling assumptions, ensuring the validity of our conclusions under various plausible scenarios.
