# Supplementary material for: Digital AVATAR therapy for distressing voices in psychosis: the phase 2/3 AVATAR2 trial
Source: Nat Med. 2024 Oct 28;30(12):3658–68. doi: 10.1038/s41591-024-03252-8 (PMC11645260; doi:10.1038/s41591-024-03252-8)
Supplement: Supplementary file 2 — Reporting Summary [file 41591_2024_3252_MOESM2_ESM.pdf]

nature portfolio

Corresponding author(s): Thomas Ward

Last updated by author(s): Aug 6, 2024

Reporting Summary

Nature Portfolio wishes to improve the reproducibility of the work that we publish. This form provides structure for consistency and transparency in reporting. For further information on Nature Portfolio policies, see our Editorial Policies and the Editorial Policy Checklist.

Statistics

For all statistical analyses, confirm that the following items are present in the figure legend, table legend, main text, or Methods section.

n/a	Confirmed	
☐	☒	The exact sample size (*n*) for each experimental group/condition, given as a discrete number and unit of measurement
☐	☒	A statement on whether measurements were taken from distinct samples or whether the same sample was measured repeatedly
☐	☒	The statistical test(s) used AND whether they are one- or two-sided<br>*Only common tests should be described solely by name; describe more complex techniques in the Methods section.*
☐	☒	A description of all covariates tested
☐	☒	A description of any assumptions or corrections, such as tests of normality and adjustment for multiple comparisons
☐	☒	A full description of the statistical parameters including central tendency (e.g. means) or other basic estimates (e.g. regression coefficient) AND variation (e.g. standard deviation) or associated estimates of uncertainty (e.g. confidence intervals)
☐	☒	For null hypothesis testing, the test statistic (e.g. *F*, *t*, *r*) with confidence intervals, effect sizes, degrees of freedom and *P* value noted<br>*Give P values as exact values whenever suitable.*
☒	☐	For Bayesian analysis, information on the choice of priors and Markov chain Monte Carlo settings
☐	☒	For hierarchical and complex designs, identification of the appropriate level for tests and full reporting of outcomes
☐	☒	Estimates of effect sizes (e.g. Cohen's *d*, Pearson's *r*), indicating how they were calculated

Our web collection on statistics for biologists contains articles on many of the points above.

Software and code

Policy information about availability of computer code

Data collection	An online data collection system for clinical trials (MACRO; InferMed Ltd, Version 4.0) was used for data entry and storage. This is hosted on a dedicated server at King's College London (KCL) and managed by the KCL Clinical Trial Unit.
Data analysis	Data description and the inferential analysis were performed using Stata version 18.0. For visualising the data, R version 4.3.3 and the ggplot2 package version 3.5.0 were utilised.

For manuscripts utilizing custom algorithms or software that are central to the research but not yet described in published literature, software must be made available to editors and reviewers. We strongly encourage code deposition in a community repository (e.g. GitHub). See the Nature Portfolio guidelines for submitting code & software for further information.

Data

Policy information about availability of data

All manuscripts must include a data availability statement. This statement should provide the following information, where applicable:

- Accession codes, unique identifiers, or web links for publicly available datasets
- A description of any restrictions on data availability
- For clinical datasets or third party data, please ensure that the statement adheres to our policy

Open access information on the AVATAR2 trial such as the trial protocol and statistical analysis plan, including example analysis code, is published in the ISRCTN registry with the identifier ISRCTN55682735; the final trial protocol (V1.2) was also published in Trials 11. Individual participant data have been deposited in King's

Open Research Data System (KORDS), but access is restricted due to privacy reasons and general data protection regulations and can only be accessed after review. The data will be made accessible following the publication of this paper. A request can be made by academic or clinical researchers to <research.data@kcl.ac.uk> for the purpose of conducting non-commercial ethically approved research. The research data team will review the request against the conditions set out in the Data Access Agreement. An initial response to requests will be formulated within a month. A Data Access Agreement will be drawn up before data can be shared.

Research involving human participants, their data, or biological material

Policy information about studies with human participants or human data. See also policy information about sex, gender (identity/presentation), and sexual orientation and race, ethnicity and racism.

Reporting on sex and gender

In compliance with the reporting requirements for sex and gender in clinical trials, this trial collected gender data based on self-reporting from participants, ensuring an inclusive approach to gender identity. Therapy completion rates have been reported in a disaggregated manner by gender. Furthermore, we conducted a moderation analysis to assess the impact of gender as a moderating variable on the effectiveness of the therapy.

Reporting on race, ethnicity, or other socially relevant groupings

In line with the standards for reporting on demographics in clinical trials, this study has collected data on participants' ethnicity and assessed socio-economic status using the Index of Multiple Deprivation (IMD). We have reported outcomes related to therapy completion rates in a disaggregated manner by ethnicity. Additionally, a moderation analysis was performed, with ethnicity and IMD Quintile serving as moderators.

Population characteristics

Our study systematically collected demographic population characteristics as presented in Table1 within the manuscript. We investigated the specific hypothesis that greater baseline complexity of voice characterisation will moderate the treatment effects of AVATAR-Brief and AVATAR-Extended compared to TAU. The following measures of baseline clinical and cognitive characteristics were also considered as potential moderators of treatment effects: PSYRATS-AH distress, Trama related symptoms: PTSD (Post-Traumatic Stress Disorder), DSO (Disturbances in Self-Organisation), Negative Symptoms (Clinical Assessment Interview for Negative Symptoms - motivation and pleasure, and expressive, CAINS MAP, CAINS EXP), duration of mental health services (early vs. not early), duration of hearing voices, age voice started, attachment (Relationship Questionnaire). We also examined demographic variables as moderators: age, gender and self-defined ethnicity.

Recruitment

Participants were recruited between 1st January 2021 and 30th November 2022, across four UK main University trial sites: the Institute of Psychiatry, Psychology & Neuroscience (King's College London), University College London, the University of Manchester and the University of Glasgow. Recruitment was conducted via referrals from clinicians at mental health services based within two (National Health Service (NHS)) providers per site, ensuring a diverse sample with respect to demography and geography. The four main NHS recruitment sites were South London and Maudsley NHS, North East London NHS Foundation Trust, Greater Manchester Mental Health NHS Foundation Trust and NHS Greater Glasgow & Clyde. The four additional NHS trusts were: Oxleas NHS Foundation Trust, Camden & Islington NHS Foundation Trust, Pennine Care NHS Foundation Trust and NHS Lanarkshire. The recruitment process was as follows: Participants were identified through close liaison with clinical staff based across specialist mental health services (inpatient and outpatient settings) in the NHS Trusts. Settings (and how these were named) varied across sites but included Early Intervention Psychosis Teams, Community Mental Health Teams (CMHTs), Rehabilitation and Recovery teams etc. After clinical staff had confirmed that a potential participant was suitable to be approached (i.e. meets study criteria and no clinical contra-indications), research workers met each potential participant to discuss the study, provide written information and time to consider it, respond to questions and seek written informed consent. Other routes to participation included contact through institutional research registers, or self-referral.

With 642 individuals assessed for eligibility and 345 successfully enrolled and randomized into three parallel study arms, our recruitment strategy was designed to minimize any potential self-selection or other biases. We have thoroughly reviewed our recruitment process and do not identify any self-selection biases or other biases that could impact the findings.

Ethics oversight

The study received ethical approval (Camberwell St. Giles Research Ethics Committee: (20/LO/0657; IRAS (Integrated Research Application System) 277118) and was prospectively registered with the ISRCTN registry at which the published trial protocol (11) and statistical analysis plan can also be accessed (ISRCTN55682735).

Note that full information on the approval of the study protocol must also be provided in the manuscript.

Field-specific reporting

Please select the one below that is the best fit for your research. If you are not sure, read the appropriate sections before making your selection.

☒ Life sciences ☐ Behavioural & social sciences ☐ Ecological, evolutionary & environmental sciences

For a reference copy of the document with all sections, see nature.com/documents/nr-reporting-summary-flat.pdf

Life sciences study design

All studies must disclose on these points even when the disclosure is negative.

Sample size

We powered the study to detect plausible effect sizes based on our previous AVATAR therapy trial (8) There we found a clinically meaningful reduction in PSYRATS-AH distress of 4.8 points, with an effect size of approximately d=0.8, but we conservatively reduced this for the current trial, to take into consideration the increase in number of centres, the follow-up comparison (not only end of treatment) and a more pragmatic trial design. We are accounting for two formal comparisons: AV-EXT vs. TAU – plausible effect size 0.6; and AV-BRF vs. TAU – plausible effect size 0.5. The study was powered for an overall treatment effect at a 5% significance level, accounting for 2 multiple

	comparisons in which the tests are correlated (at r=0.5), giving an alpha level for each test of 0.035. Accordingly, a sample size of 92 per group or 276 in total in the analysis set had 90% power to detect a minimum clinically significant difference (effect size) of 0.5 standard deviations. We sought to recruit 345 participants in total at baseline (87 per site), with n=115 per treatment arm, allowing for conservative attrition rates of 20%.
Data exclusions	The primary analyses were carried out using the intention to treat sample: participants were analysed in the group they are randomised to, and available data from all participants is included, including those who do not complete therapy.
Replication	This clinical trial was not a replication study. However it was designed and delivered using an approach which supports future replication.
Randomization	After baseline assessment, we randomly assigned (1:1:1) eligible participants, via a secure independent web-based service hosted by King's Clinical Trials Unit, using randomly varying sized blocks (3 and 6), stratified by site and baseline voice characterisation (more/less) as defined by meeting the threshold for more highly characterised voices (score>7) on the Voice Characterisation Checklist (27).
Blinding	Research assessors were masked to allocation, and procedures were followed to maintain their masking (assessors did not have access to clinical records after the baseline (pre-randomisation) assessment or access to the therapy database at any stage), all assessments were done at sites remote from the clinic, and participants were reminded before each assessment not to disclose their allocation. It is not possible to mask psychological therapy participants or therapists to their allocation; site co-ordinators were unmasked and informed participants. Therapists were allocated at each site based on availability. Breaks in assessor masking were recorded, and if unmasking occurred, re-allocation to another rater occurred. All primary and key secondary outcomes (PSYRATS-AH scale) were assessed by blinded assessors. Unmasking occurred in 29 people (8.4%) at 16-weeks and 15 people (4.3%) at 28-weeks. All assessments of these people were scored by blinded assessors following these instances of unmasking.

Reporting for specific materials, systems and methods

We require information from authors about some types of materials, experimental systems and methods used in many studies. Here, indicate whether each material, system or method listed is relevant to your study. If you are not sure if a list item applies to your research, read the appropriate section before selecting a response.

Materials & experimental systems

n/a	Involved in the study
☒	☐ Antibodies
☒	☐ Eukaryotic cell lines
☒	☐ Palaeontology and archaeology
☒	☐ Animals and other organisms
☐	☒ Clinical data
☒	☐ Dual use research of concern
☒	☐ Plants

Methods

n/a	Involved in the study
☒	☐ ChIP-seq
☒	☐ Flow cytometry
☒	☐ MRI-based neuroimaging

Clinical data

Policy information about clinical studies

All manuscripts should comply with the ICMJE guidelines for publication of clinical research and a completed CONSORT checklist must be included with all submissions.

Clinical trial registration	ISRCTN55682735
Study protocol	The trial protocol is published in the ISRCTN registry with the identifier ISRCTN55682735; the final trial protocol prior to recruitment (V1.2) was also published in Trials journal.
Data collection	Between 1st January 2021 and 30th November 2022, we assessed 642 people for eligibility, recruiting 345 participants. Participants were randomised at four study sites, each recruiting from two mental health service providers, in the United Kingdom (3 England: South London, North London, Manchester; one Scotland: Glasgow) and were randomly allocated to three parallel arms: 116 to AV-BRF, 114 to AV-EXT and 115 to TAU control. Participants were referred by a clinician in the participating clinical sites. Other routes to participation included contact through institutional research registers, or self-referral.<br><br>The four UK main University trial sites: the Institute of Psychiatry, Psychology & Neuroscience (King's College London), University College London, the University of Manchester and the University of Glasgow. The four main NHS recruitment sites were South London and Maudsley NHS, North East London NHS Foundation Trust, Greater Manchester Mental Health NHS Foundation Trust and NHS Greater Glasgow & Clyde. The four additional NHS trusts were: Oxleas NHS Foundation Trust, Camden & Islington NHS Foundation Trust, Pennine Care NHS Foundation Trust and NHS Lanarkshire.<br><br>Participants were identified through close liaison with clinical staff based across specialist mental health services (inpatient and outpatient settings) in the NHS Trusts. Settings (and how these were named) varied across sites but included Early Intervention Psychosis Teams, Community Mental Health Teams (CMHTs), Rehabilitation and Recovery teams etc.
Outcomes	The pre-specified primary outcome for the study was reduction in distress associated with voices at end of treatment (16weeks) and follow up (28weeks), as measured by the distress dimension of the Psychotic Symptoms Rating Scale (PSYRATS-AH) (5 items, distress (2 items), negative content (2 items) and control. The PSYRATS-AH is a dimensional semi-structured assessor-rated clinical interview

assessing auditory hallucinations, comprising in total 11 items, each item scored from 0 (voices not present) to 4.
Key secondary outcomes, as specified in the primary hypotheses, were reductions in the voice frequency scale score (3 items: frequency, duration, and disruption items) and the total severity score (all 11 items) on the PSYRATS-AH scale at 16 and 28 weeks.
Other secondary outcomes were a mix of assessor-rated and self-reported measures, with effects estimated at 16 and 28 weeks. These included distressing beliefs (PSYRATS-Delusions), Wellbeing (Warwick-Edinburgh Mental Wellbeing Scale (WEMWBS), Psychological recovery (Choice of Outcome in CBT for Psychosis (CHOICE),Fearful attachment (Relationships Questionnaire Item), Voices Action and Acceptance Scales (VAAS), measuring acceptance-based attitudes and actions in relation to voice-hearing experiences, Mood (Depression, Anxiety and Stress Scales (DASS), and Beck Depression Inventory, Anxiety in daily life (using Experience Sampling Measure), Voice power (Voice Power Differential Scale item) and Beliefs about Voices Questionnaire (omnipotence, malevolence and benevolence, total , BAVQ-R), and Trauma-related symptoms (International Trauma Questionnaire) (16 weeks only).
Clinical characteristics of participants were further assessed at baseline with the Clinical Assessment Interview for Negative Symptoms (CAINS) and Scale for Assessment of Positive Symptoms (SAPS). (Further details of all measures are provided in Supplementary Materials)

Plants

Seed stocks

N/A

Novel plant genotypes

N/A

Authentication

N/A
